# Endothelial cells are progenitors of cardiac pericytes and vascular smooth muscle cells

**DOI:** 10.1038/ncomms12422

**Published:** 2016-08-12

**Authors:** Qi Chen, Hui Zhang, Yang Liu, Susanne Adams, Hanna Eilken, Martin Stehling, Monica Corada, Elisabetta Dejana, Bin Zhou, Ralf H. Adams

**Affiliations:** 1 Max Planck Institute for Molecular Biomedicine, Department of Tissue Morphogenesis, Faculty of Medicine, University of Münster, D-48149 Münster, Germany; 2 Key Laboratory of Nutrition and Metabolism, Institute for Nutritional Sciences, Shanghai Institutes for Biological Sciences, Chinese Academy of Sciences, Shanghai 200031, China; 3 Electron Microscopy and Flow Cytometry Units, Max Planck Institute for Molecular Biomedicine, D-48149 Münster, Germany; 4 IFOM Fondazione, FIRC Institute of Molecular Oncology, 20139 Milan, Italy; 5 Department of Biosciences, University of Milan, 20133 Milan, Italy

## Abstract

Mural cells of the vessel wall, namely pericytes and vascular smooth muscle cells, are essential for vascular integrity. The developmental sources of these cells and molecular mechanisms controlling their progenitors in the heart are only partially understood. Here we show that endocardial endothelial cells are progenitors of pericytes and vascular smooth muscle cells in the murine embryonic heart. Endocardial cells undergo endothelial–mesenchymal transition and convert into primitive mesenchymal progenitors expressing the platelet-derived growth factor receptors, PDGFRα and PDGFRβ. These progenitors migrate into the myocardium, differentiate and assemble the wall of coronary vessels, which requires canonical Wnt signalling involving Frizzled4, β-catenin and endothelial cell-derived Wnt ligands. Our findings identify a novel and unexpected population of progenitors for coronary mural cells with potential relevance for heart function and disease conditions.

## Abstract (web summary)

Pericytes and vascular smooth muscle cells are crucial for functional blood vessels, but the developmental sources of these cells are incompletely understood. Here, the authors show that endocardial endothelial cells give rise to cardiac mural cells, which are controlled by Wnt signalling.

## Graphical abstract

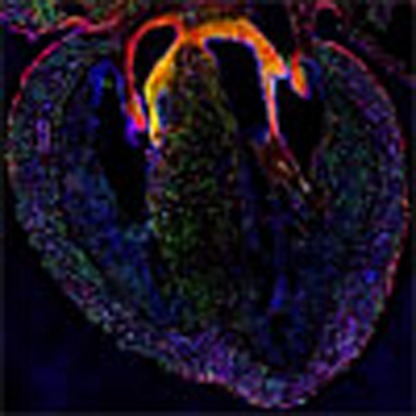

Ischaemic heart disease, which is typically caused by dysfunction of the coronary vasculature, is the leading cause of death worldwide^1^,^2^. The integrity, perfusion and function of blood vessels inside and outside of the heart critically rely on the interaction of different cell types^3^,^4^,^5^,^6^. While a monolayer of endothelial cells (ECs) encloses the vessel lumen, mural cells, namely pericytes, are associated with the abluminal surface of capillaries. Vascular smooth muscle cells (vSMCs), that is, mural cells covering larger calibre arteries and veins, are thought to be closely related to pericytes and, in heart, are even derived from pericytes^7^,^8^,^9^. Mural cells stabilize vessels through physical and molecular interactions with adjacent ECs, and absence of mural cells leads to vascular leakage and haemorrhaging^3^,^4^,^7^. Pericytes and their progenitors have high clinical relevance and, accordingly, several studies have explored the potential of these cells for cardiac regeneration and heart tissue engineering^10^,^11^,^12^,^13^,^14^,^15^. Remarkably, mural cells expressing the markers platelet-derived growth factor receptor β (PDGFRβ), CD146 and NG2/Cspg4 have been proposed to function as mesenchymal stem cells in multiple organs and act as myofibroblast progenitors during injury-induced fibrosis^16^,^17^,^18^.

Despite the great importance of mural cells, the precise properties and developmental sources of these cells remain poorly understood. In the heart, previous studies have shown that progenitor cells derived from the embryonic epicardium invade into the myocardium and give rise to cardiomyocytes and mural cells^19^,^20^,^21^. It was also shown that these cardiac mural cell progenitors express PDGFRβ and require PDGFRβ-driven phosphoinositide 3′ kinase (PI3K) signalling for their migration^21^. In addition to PDGFRβ, the related receptor PDGFRα is expressed by epicardial cells. Combined tissue-specific inactivation of the genes for both PDGF receptors disrupted the migration of epicardial progenitors into the myocardium, while it had no effect on the proliferation or survival of these cells. Furthermore, it was also shown that PDGFRα is specifically required for the formation of cardiac fibroblast, whereas only PDGFRβ is indispensable for mural cell development^22^. However, genetic lineage tracing indicated that not all cardiac mural cells are derived from epicardial cells^19^,^20^,^21^. Likewise, inactivation of the *Pdgfrb* gene (encoding PDGFRβ) in epicardial cells did not eliminate all cardiac mural cells^21^ arguing for additional, so far unknown developmental sources of pericytes and vSMCs in the heart.

In this study, we have identified endocardial ECs as novel progenitors for mural cells in the heart with the help of genetic lineage tracing and gene inactivation experiments. While endothelial and mural cells belong to distinct lineages in most tissues and model systems, our work also establishes that this separation is not maintained in the developing cardiac vasculature. Thus, mural and endothelial cells develop from a common progenitor population during early stages of heart development.

## Results

### Molecular markers of cardiac mural cells

As mural cells are known to show heterogeneous expression of molecular markers^7^, we first characterized mural cells in sections of murine heart at postnatal day (P) 6. In these experiments, *Tg(Cspg4-dsRed.T1)*^*1Akik*^ reporter mice were used to identify the *in vivo* expression pattern of NG2. In *Pdgfra*^*tm11(EGFP)Sor*^ knockin reporter mice, PDGFRα expression is detected via a nuclear green fluorescent protein (H2B-GFP) reporter. PDGFRβ+ cells and their progeny were stably labelled with *Pdgfrb(BAC)-CreERT2* transgenic mice, which were recently generated by our group. These mouse lines (Supplementary Table 1) in combination with immunostaining showed that the majority of mural cells associated with coronary capillaries were positive for platelet-derived growth factor receptor β (PDGFRβ) and the proteoglycan NG2 but lacked PDGFRα expression (Supplementary Fig. 1a–e). Only few cardiac mural cells expressed CD13 or desmin (Supplementary Fig. 1d,f), which have been used as pericyte markers in other organs. Desmin was also prominently expressed by cardiomyocytes (Supplementary Fig. 1f). On the basis of this analysis, we defined capillary-associated mural cells as PDGFRβ+ NG2+ PDGFRα- cells.

### Identification of putative cardiac mural cell progenitors

**Figure 1:**
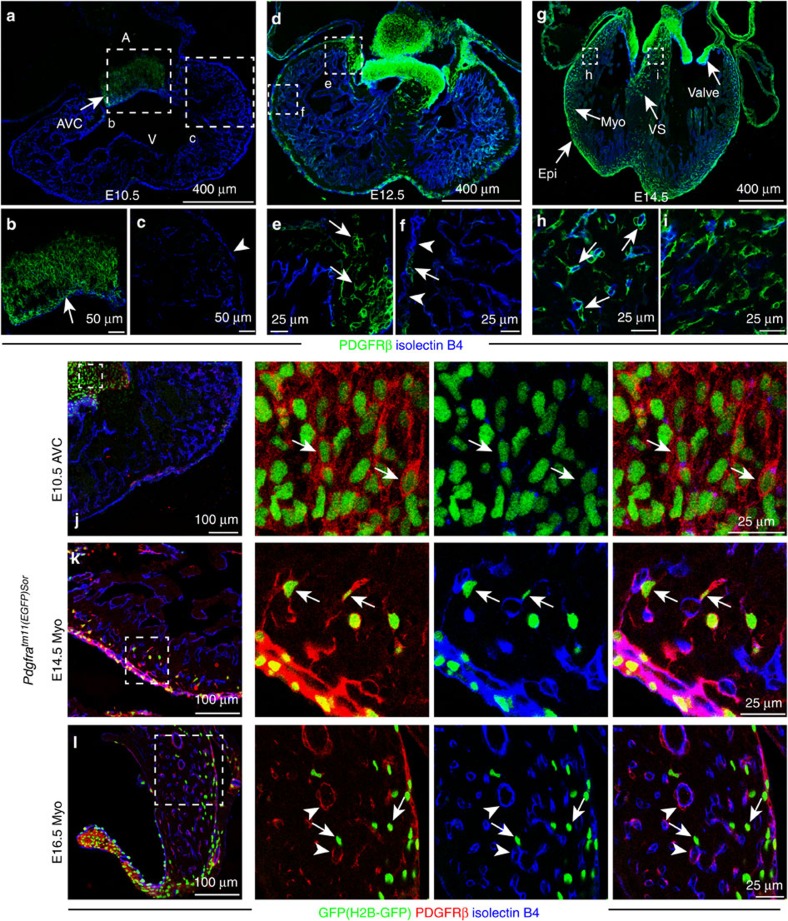
Developmental distribution and molecular properties of PDGFRβ+ cells. (**a**–**i**) Distribution of PDGFRβ (green) immunostained cells from E10.5 to E14.5. Heart sections from wild-type mice were stained for PDGFRβ (green) and isolectinB4 (blue). Arrows indicate PDGFRβ+ cells, arrowheads mark PDGFRβ- epicardial cells at the indicated stages. Panels at the bottom (**b**,**c**,**e**,**f**,**h**,**i**) are higher magnifications of insets in (**a**,**d**,**g**), respectively. A, atrium; V, ventricle; AVC, atrioventricular canal; Epi, epicardium; Myo, myocardium; VS, ventricular septum. (**j**–**l**) Transient co-expression of PDGFRβ (red) and PDGFRα (*Pdgfra*^tm11(EGFP)Sor^ reporter; nuclear H2B-GFP; green) during heart development. ECs, isolectinB4 (blue). Arrows indicate GFP+ PDGFRβ+ double-positive cells in the E10.5 AVC (**j**) and E14.5 myocardium (**k**). Double-positive cells were very rare in the E16.5 myocardium (**l**), whereas GFP- PDGFRβ+ mural cells (arrowheads) and GFP+ PDGFRβ- interstitial cells (arrows in **l**) were abundant.

In contrast to postnatal heart, PDGFRβ+ cells at midgestation were not associated with myocardial capillaries, but were instead confined to large clusters located in atrioventricular canal (AVC) and outflow tract (OFT; Fig. 1a–c; Supplementary Fig. 2a). Expression of PDGFRβ protein was absent in epicardial cells at embryonic day (E) 10.5, and, likewise, PDGFRβ expression was not detectable in cells of the proepicardial organ at E9.5 (Supplementary Fig. 2a). In addition to the large clusters in the AVC and heart valves, some PDGFRβ+ cells were detected in the myocardium, ventricular septum and developing valves at E12.5 (Fig. 1d–f). From E14.5, PDGFRβ+ cells were abundant in myocardium and closely associated with ECs (Fig. 1g–i).

We further characterize the expression pattern of PDGFRα and NG2 in PDGFRβ+ cells during this dynamic developmental process. At E10.5, cells in the AVC also showed expression of PDGFRα (that is, nuclear GFP signal in the *Pdgfra*^*tm11(EGFP)Sor*^ reporter; Fig. 1j) but were negative for NG2/Cspg4 suggesting that these were primitive mesenchymal cells (Supplementary Fig. 2b). PDGFRβ+ cells in the valves continued to co-express PDGFRα and lacked NG2 even in postnatal animals (Supplementary Fig. 2c). In contrast, the PDGFRβ+ cells that were located around coronary blood vessels in the compact myocardium at E14.5 (Fig. 1g–i) were only partially positive for PDGFRα but showed expression of NG2 (Fig. 1k; Supplementary Fig. 2b). Quantitation showed that 71.5% of the PDGFRα+ (128/179) and 100% of the NG2+ (166/166) cells in compact myocardium were positive for PDGFRβ, and these cells were typically found in direct proximity of capillaries (Fig. 1k; Supplementary Fig. 2b). At E16.5, PDGFRβ+ PDGFRα+ double-positive cells were very rarely visible in the myocardium, whereas PDGFRβ+ NG2+ PDGFRα− cells were abundant around vessels and therefore showed the same morphological features and marker expression as postnatal pericytes (Fig. 1l; Supplementary Fig. 2b). Altogether, these data argued for the existence of primitive mesenchymal progenitors in the embryonic AVC and OFT, which can migrate into the myocardium and differentiate into cardiac mural cells.

### Genetic fate mapping of mural cell progenitors

**Figure 2:**
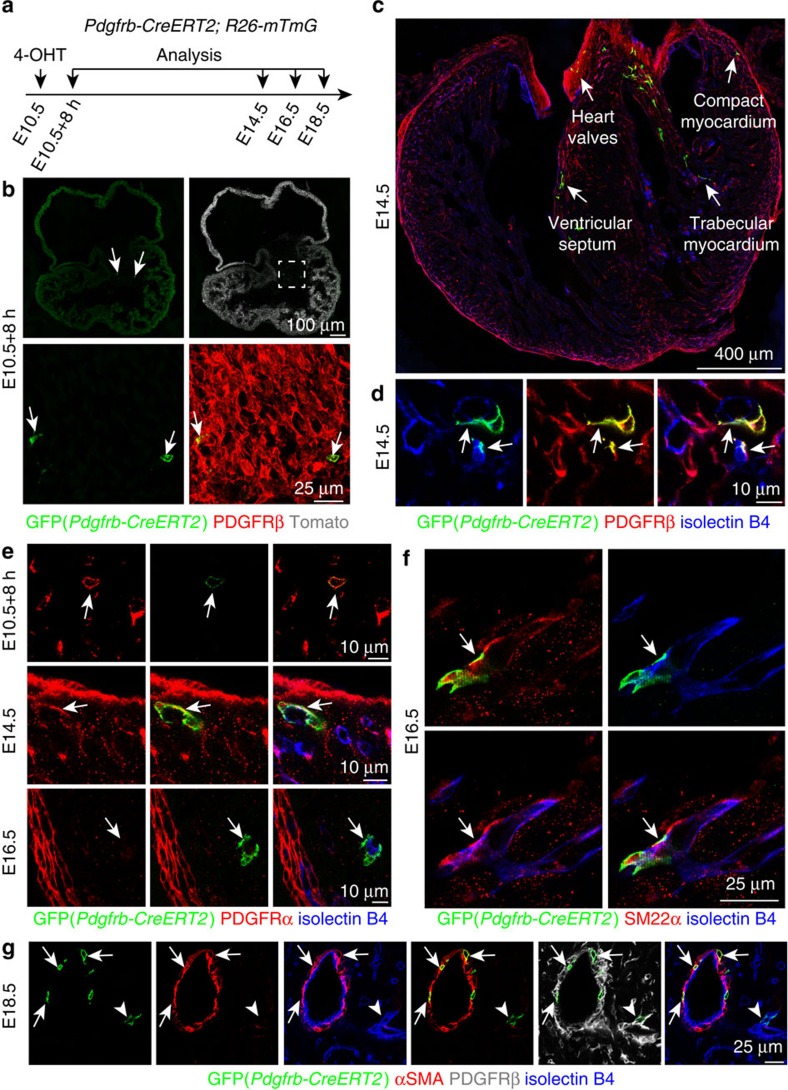
Cardiac mural cells originate from mesenchymal progenitors. (**a**–**g**) Clonal analysis using *Pdgfrb(BAC)-CreERT2 Rosa26-mTmG* mice. (**a**) Experimental strategy indicating the stages of 4-hydroxytamoxifen (4-OHT) administration and analysis. (**b**) GFP+ cells (green, arrows) in AVC at 8 hours after 4-OHT administration. PDGFRβ (red) immunostaining and unrecombined cells (tdTomato, white/red) are shown. (**c**,**d**) Overview of GFP+ cell distribution in the indicated regions of E14.5 heart (**c**). At higher magnification, GFP+ PDGFRβ+ cells (arrows) in the myocardium were found in association with isolectin B4-labelled vessels (blue) (**d**). (**e**) Arrows indicate representative GFP+ cells, which were PDGFRα+ (red) in the E10.5 AVC and E14.5 myocardium, but PDGFRα- and located at isolectin B4+ vessels (blue) in E16.5 myocardium. (**f**) *Pdgfrb(BAC)-CreERT2*-labelled cell clones (GFP, green) gave rise to SM22α+ mural cells (red, arrow) at E16.5. ECs, isolectin B4 (blue). (**g**) *Pdgfrb(BAC)-CreERT2*-marked cell clones (GFP, green) were identified as αSMA+ vSMCs (red, arrows) at E18.5. Arrowheads mark a αSMA- perivascular cells. ECs, isolectin B4 (blue).

To test directly whether cardiac pericytes and vSMCs were generated from PDGFRβ+ mesenchymal progenitors in the embryonic AVC/OFT, we used *Pdgfrb(BAC)*−*CreERT2* transgenic mice to genetically label these cells and their descendants. Immunostaining of the oestrogen receptor domain (ERT2) in the CreERT2 fusion protein labelled cells in the AVC and OFT, but not in epicardium at E10.5 (Supplementary Fig. 3a–h), which was consistent with the pattern of PDGFRβ expression reported above (Fig. 1a). In contrast, no anti-ERT2 immunosignals were detected in Cre-negative mice (Supplementary Fig. 3a,e). These results indicated that *Pdgfrb(BAC)-CreERT2* transgenic mice enable genetic fate mapping of PDGFRβ+ mesenchymal progenitors in the embryonic AVC and OFT. For this purpose, *Pdgfrb(BAC)-CreERT2* mice were interbred with *Rosa26-mTmG* Cre reporter transgenic animals. The resulting mice displayed ubiquitous expression of membrane-anchored tdTomato fluorescent protein, while activation of Cre recombinase led to an irreversible switch to membrane-anchored GFP expression in Cre-expressing cells and their progeny (Supplementary Table 1). Single administration of a low dose of 4-hydroxytamoxifen (4-OHT) at E10.5 led to rare recombination events, which were readily detectable 8 h later as isolated GFP+ PDGFRβ+ PDGFRα+ NG2– mesenchymal cells (Fig. 2a,b,e; Supplementary Fig. 4a,b). GFP+ cells at this stage were found exclusively in the AVC or OFT (85 cells from 9 mice, 44 in AVC, 41 in OFT). Descendants of this initially labelled population showed co-expression of PDGFRα and NG2 at E14.5, whereas GFP+ PDGFRα− NG2+ cells were associated with vessels in the compact myocardium as well as in the ventricular septum at E16.5 (Fig. 2c–e; Supplementary Fig. 4b). The morphology, marker expression and vessel association of GFP+ cells indicated that they had differentiated into pericytes. Larger calibre vessels at E16.5 were also surrounded by GFP+ SM22α+ cells, indicating that the initially labelled cell population can also give rise to vSMCs (Fig. 2f). This is further supported by the presence of GFP+ and α–smooth muscle actin+ (αSMA) cells around coronary arteries (Fig. 2g). Furthermore, the increasing distance of GFP+ cells from the original PDGFRβ+ clusters (Supplementary Fig. 4c) supported the concept that primitive mesenchymal progenitor cells had migrated from the AVC/OFT into other regions of the heart before they became associated with vessels as pericytes or vSMCs. While the distribution of GFP+ cells was widespread, their abundance was highest in the top part of the ventricular septum (Supplementary Fig. 4d,e). The spatial distribution of GFP+ cells also indicated that they were unlikely to be derived from epicardium, which was previously identified as a source of mural cells^19^,^20^. These data support that cardiac pericytes and vSMCs are derived from PDGFRβ+ PDGFRα+ NG2− progenitors in the AVC/OFT during heart development.

### Endothelial cells give rise to PDGFRβ+ mural cells

**Figure 3:**
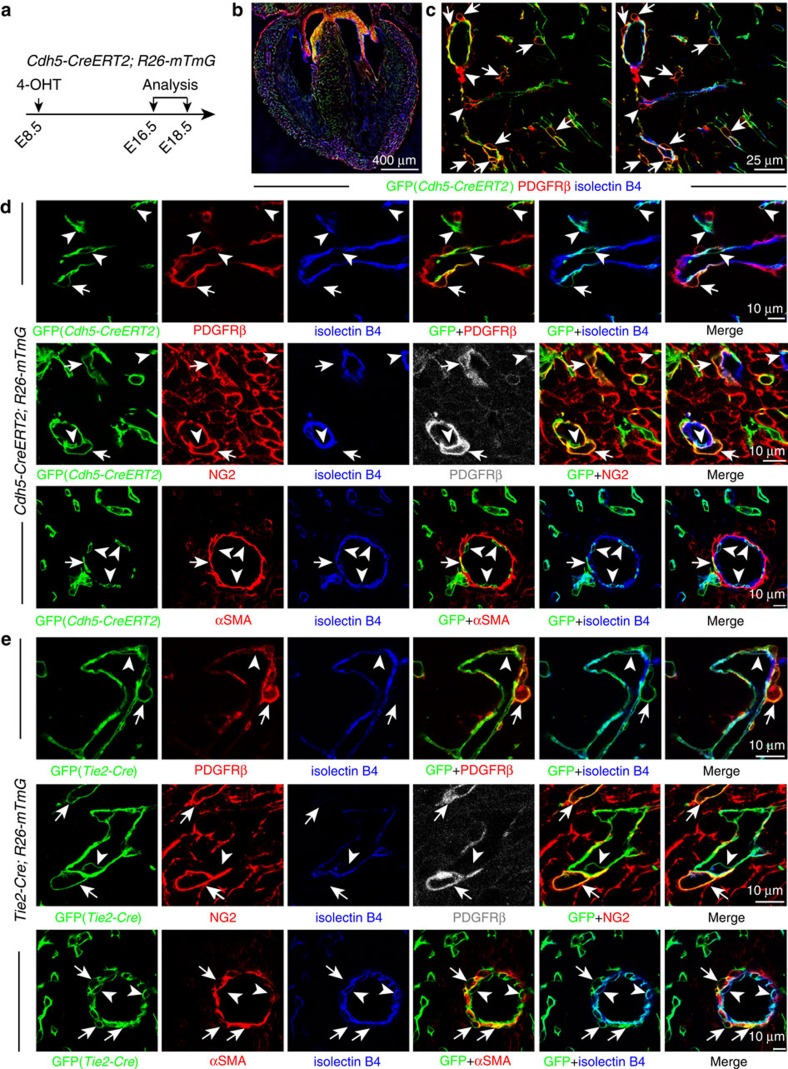
Cardiac mural cells originate from endothelial cells. (**a**–**d**) Clonal analysis of *Cdh5-CreERT2 Rosa26-mTmG* double-transgenic mice. (**a**) Clonal analysis strategy of *Cdh5-CreERT2 Rosa26-mTmG* mice indicating the stages of 4-hydroxytamoxifen (4-OHT) administration and analysis. (**b**) Overview of GFP+ cell distribution in heart at E16.5. (**c**) Images showing widespread distribution of EC-derived GFP+ PDGFRβ+ mural cells (arrows). Arrowheads mark GFP− PDGFRβ+ cells. (**d**) Higher magnification images showing vessel-associated (isolectin B4), EC-derived GFP+ PDGFRβ+ NG2+ mural cells (arrows) at E16.5 (top and middle row) and GFP+ αSMA+ mural cells (arrows) at E18.5 (bottom row). Arrowheads mark GFP+ (green) isolectin B4+ (blue) ECs. Grey channel was not included in merged image. (**e**) Lineage tracing in *Tie2-Cre Rosa26-mTmG* double-transgenic hearts. Confocal images showing GFP+ PDGFRβ+ NG2+ and GFP+ αSMA+ mural cells (arrows) in association with myocardial capillary ECs (isolectin B4). Arrowheads mark GFP+ (green) isolectin B4+ (blue) ECs. Grey channel was not included in merged image.

Next, we wanted to identify the developmental origin of the PDGFRβ+ mesenchymal progenitors in the AVC/OFT. Mural cells in brain and thymus have been shown to be derived from the neural crest, which also contributes to the OFT^7^,^23^,^24^. However, analysis of GFP expression in *Wnt1-Cre Rosa26-mTmG* mice, which labelled neural crest-derived cell populations, showed very little staining of the heart ventricle at E14.5 (Supplementary Fig. 5a). This suggested limited neural crest contribution to cardiac mural cells in the heart ventricle. Surprisingly, we found that PDGFRβ+ PDGFRα− NG2+ cardiac pericytes showed GFP signal in EC-specific *Cdh5-CreERT2* mice in the *Rosa26-mTmG* Cre reporter background. *Cdh5-CreERT2*-mediated recombination labelled ECs but also cardiac mural cells when 4-OHT was injected at E8.5 (Fig. 3a–d; Supplementary Fig. 5b,d). These results argued for an unexpected endothelial cell origin of cardiac pericytes. We verified these results using *Tie2-Cre* transgenic mice, another EC-specific line that is, however, constitutively active and not tamoxifen-inducible. *Tie2-Cre Rosa26-mTmG* double-transgenic mice showed GFP signal in PDGFRβ+ NG2+ cardiac pericytes in addition to the expected labelling of endothelium (Fig. 3e; Supplementary Fig. 5c and 5e). αSMA+ vSMCs also showed GFP expression in tamoxifen-inducible *Cdh5-CreERT2 Rosa26-mTmG* and constitutively active *Tie2-Cre Rosa26-mTmG* double-transgenic hearts (Fig. 3d,e). Approximate 10.75% (20/186) of all αSMA+ vSMCs expressed GFP in the *Tie2-Cre Rosa26-mTmG* background. Altogether, these results established that ECs in the heart differentiate into mural cells during embryonic development.

**Figure 4:**
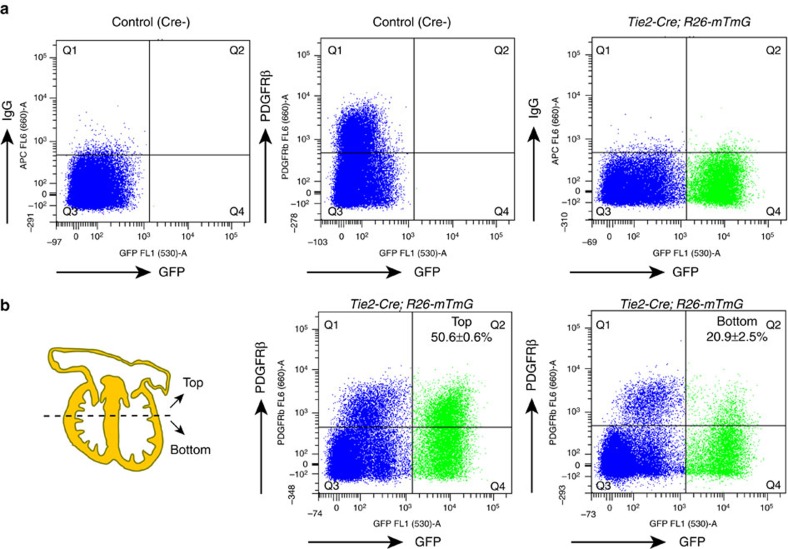
Contribution of endothelial cells to cardiac mural cells. (**a**,**b**) Flow cytometry analysis of cardiac PDGFRβ+ cells of endothelial cell origin at E14.5. (**a**) Littermate *Tie2-Cre*-negative mice, IgG-APC and PDGFRβ-APC antibodies were used to define the gating of signals. (**b**) The top and bottom of hearts were analysed independently. Numbers indicate percentage of GFP+ PDGFRβ+ cells relative to total PDGFRβ+ population in top or bottom parts of heart, respectively. *n*=3 (4 or 5 hearts/experiment). Error bars±s.e.m.

Given that the very close association of pericytes and ECs complicates their unambiguous identification in immunstained tissue sections, we quantitated the contribution of ECs to PDGFRβ+ cardiac mural cells by flow cytometric analysis of *Tie2-Cre Rosa26-mTmG* double-transgenic hearts. Cre- littermate and IgG isotype antibody controls were used to define the fraction of GFP+ (EC-derived) PDGFRβ+ cells (Fig. 4a). To exclude the contribution of PDGFRβ+ mesenchymal cells in heart valves, we manually separated hearts into two parts before sample dissociation. Flow cytometry showed that the ratio of GFP+ PDGFRβ+ cells relative to all PDGFRβ+ cells was 50.6±0.6% in the top part including heart valves, while 20.9±2.5% of PDGFRβ+ cells was positive for GFP in the bottom part (mainly consisting of ventricular wall and septum; Fig. 4b). These data showed that a substantial portion (>20%) of mural cells in the developing heart have an endothelial origin.

Furthermore, we isolated EC-derived mesenchymal progenitors from the AVC/OFT to test their differentiation potential *ex vivo* (Supplementary Fig. 6a). Consistent with the results obtained *in vivo*, this population gave rise to cells expressing mural markers including NG2, desmin and αSMA (Supplementary Fig. 6b–d). In contrast, no adipogenic and osteogenic differentiation was obtained in culture (Supplementary Fig. 6e,f). These results prove that EC-derived mesenchymal progenitors have the potential to differentiate into pericytes and vSMCs both *in vivo* and *ex vivo*.

### Endocardial ECs are the source of cardiac mural cells

**Figure 5:**
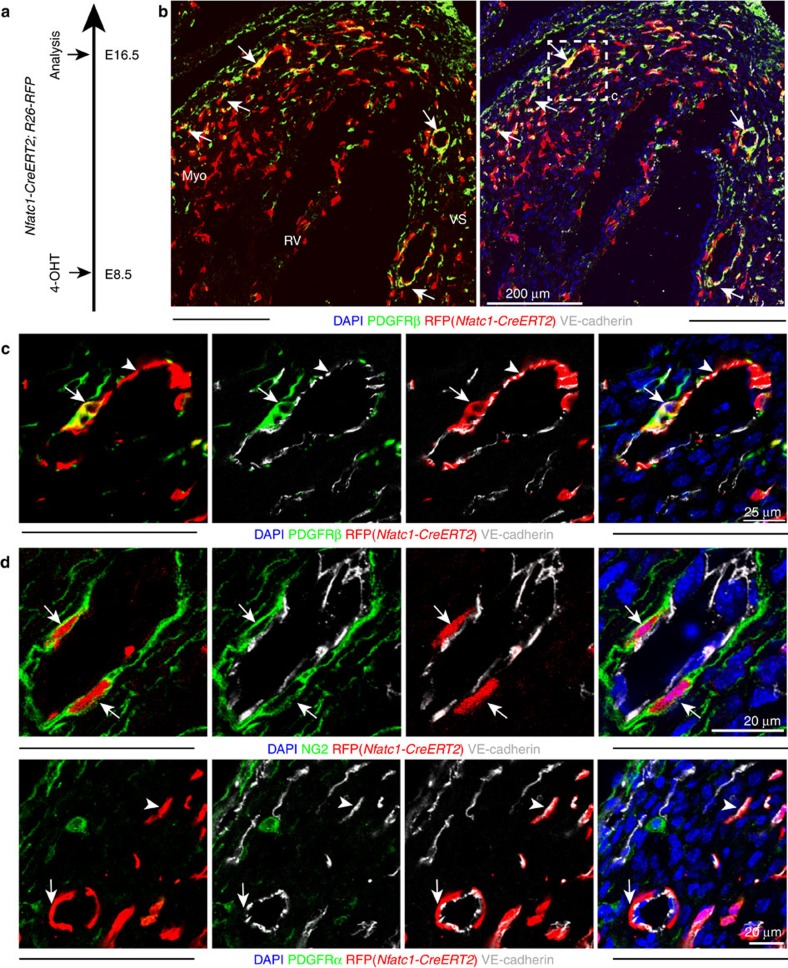
Cardiac mural cells originate from endocardial endothelial cells. (**a**) Clonal analysis strategy of *Nfatc1-CreERT2 Rosa26-RFP* mice indicating the stages of 4-hydroxytamoxifen (4-OHT) administration and analysis. (**b**) At E16.5, RFP+ PDGFRβ+ cells were found in ventricular septum and compact myocardium (arrows). VS, ventricular septum; Myo, myocardium; RV, right ventricle. (**c**) Higher magnification images showing vessel-associated (VE-cadherin, white; arrowhead), endocardium-derived RFP+ (red) PDGFRβ+ (green) mural cells (arrow) in E16.5 compact myocardium. Nuclei, DAPI (blue). (**c**) is higher magnification of inset in **b**. (**d**) Clonal analysis in *Nfatc1-CreERT2 Rosa26-RFP* double-transgenic hearts at E16.5 following 4-OHT administration at E8.5. Higher magnification images show RFP+ PDGFRα- NG2+ VE-cadherin- mural cells (arrows) in association with myocardial capillary ECs.

Previous work has shown that at least two distinct EC subpopulations, which are Apln+ or Nfatc1+, contribute to the coronary endothelium^25^,^26^,^27^,^28^. The Apln+ EC subpopulation is located in the subepicardial (outer) myocardium and can be genetically labelled with *Apln-CreERT2* transgenic mice. In contrast, Nfatc1+ endocardial ECs, which line the inner surface of heart chambers, can be tracked with the *Nfatc1-CreERT2* transgene^25^,^26^,^28^. Following tamoxifen administration at E10.5, *Apln-CreERT2 Rosa26-mTmG*-labelled cells were abundant in the myocardial endothelium at E16.5. GFP signal was, however, absent in valves and in myocardial PDGFRβ+ pericytes (Supplementary Fig. 7a–d) arguing that Apln+ subepicardial ECs do not give rise to mesenchymal progenitors in the AVC/OFT or to cardiac mural cells. To test whether mural cells instead might be derived from endocardial ECs, genetic fate mapping was performed in *Nfatc1-CreERT2* mice in the *Rosa26-RFP* Cre reporter background after 4-OHT administration at E8.5. At E16.5, endocardial EC-derived RFP+ cells were found closely attached to coronary blood vessels (Fig. 5a–d). These vessel-associated cells showed expression of PDGFRβ and NG2 but not PDGFRα (Fig. 5c,d), which proved that endocardial ECs are a source of cardiac mural cells.

The findings above indicate that ECs possess heterogeneous properties during midgestation development, which enables a subpopulation of Nfatc1+ endocardial ECs to transdifferentiate into cardiac mural cells. Indeed, endocardium-derived, *Nfatc1-CreERT2*-labelled cells were found in the E10.5 AVC and showed expression of PDGFRβ and PDGFRα but not NG2 or the endothelial marker VE-Cadherin/Cdh5 (Supplementary Fig. 8a), indicating that they had undergone endothelial–mesenchymal transition^29^. Similarly, *Tie2-Cre*-labelled mesenchymal cells in the E10.5 AVC showed expression of PDGFRβ and PDGFRα but not NG2 (Supplementary Fig. 8b). As the *Pdgfrb(BAC)-CreERT2* lineage tracing data show, PDGFRβ+ mesenchymal cells migrate out of the AVC/OFT and give rise to mural cells in the myocardium (Fig. 2). Thus, the generation of EC-derived mural cells in the heart is a multistep process in which the initial endothelial–mesenchymal transition is followed by the migration into the myocardium and the differentiation into vessel-associated pericytes and vSMCs.

### Wnt signalling controls mural cell progenitor recruitment

To gain insight into the molecular processes controlling mural cell development in the heart, *Pdgfrb(BAC)-CreERT2* mice were interbred with *Rpl22*^*tm1.1Psam*^ transgenic animals, which enabled Cre recombinase-controlled RiboTag profiling^30^ of actively translating RNAs in embryonic cardiac PDGFRβ+ cells (Supplementary Table 1). This analysis showed that PDGFRβ+ cells expressed components of the Wnt signalling pathway, including the receptor Frizzled4, the signal transducer β-catenin and the downstream target Axin2 (Supplementary Fig. 9a). Further arguing for a role of Wnt signalling, β-galactosidase expression in *Tg(BAT-lacZ)*^*3Picc*^ reporter mice, which contain TCF/LEF binding sites detecting β-catenin-dependent gene expression, labelled PDGFRβ+ PDGFRα+ mesenchymal cells in E13.5 heart valves (Supplementary Fig. 9b and Supplementary Table 1).

**Figure 6:**
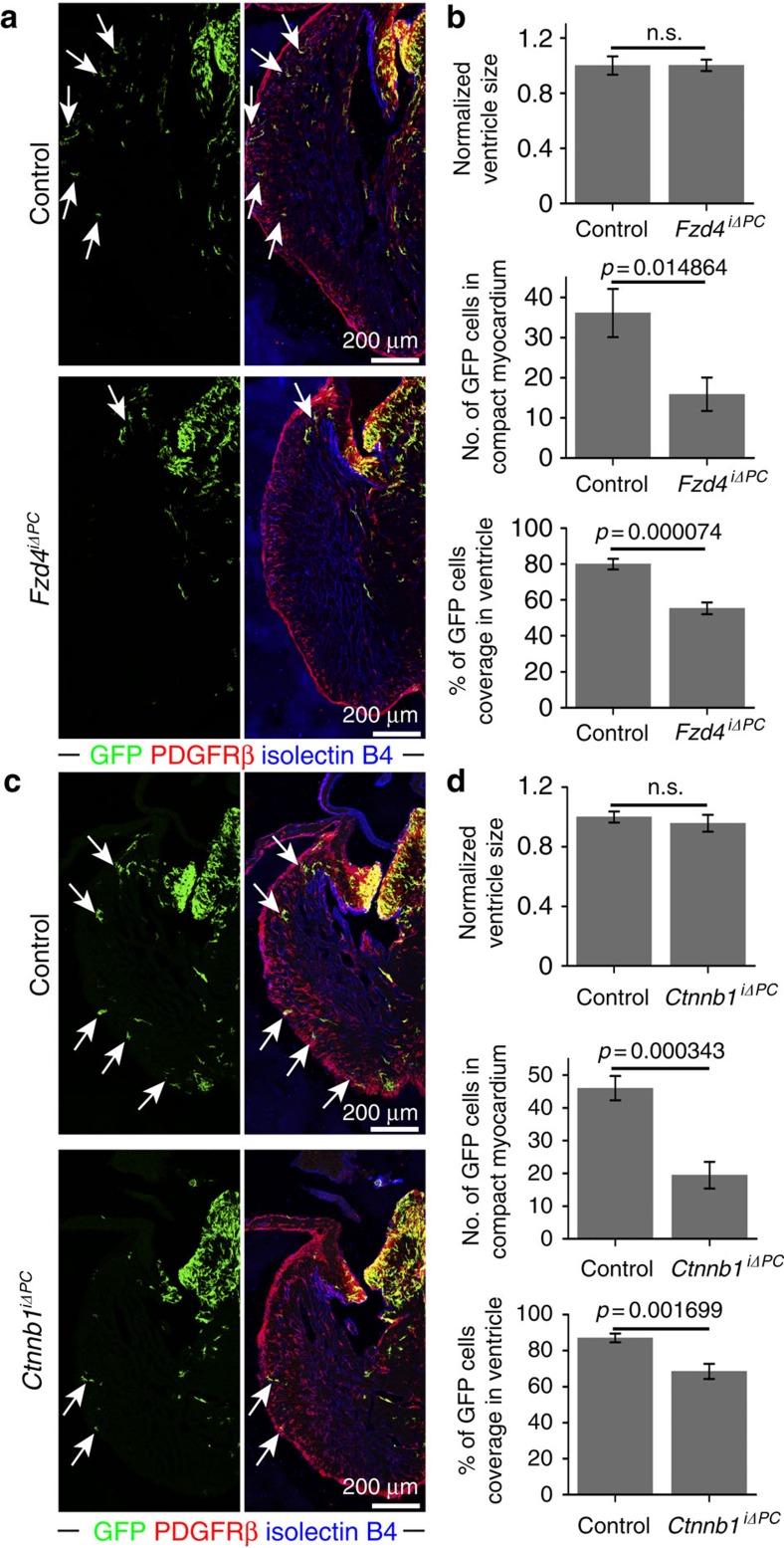
PDGFRβ+ cells require Wnt signalling for recruitment to the compact myocardium. (**a**) Maximum intensity projections showing the distribution of *Pdgfrb(BAC)-CreERT2*-labelled GFP+ cells (green; arrows) in *Fzd4*^iΔPC^ mutant and littermate control (*Pdgfrb(BAC)-CreERT2*^*+/T*^ *Fzd4*^*lox/+*^) E14.5 heart sections. Note profound reduction of GFP+ cells in *Fzd4*^iΔPC^ compact myocardium. (**b**) Statistical analysis of *Fzd4*^iΔPC^ versus control ventricle size, number of GFP+ cells in E14.5 compact myocardium and relative coverage of ventricle by GFP+ cells (*n*=8 in each group). Error bars,±s.e.m. *P* values, two-tailed unpaired *t*-test; NS, no significance. (**c**) Maximum intensity projections showing *Pdgfrb(BAC)-CreERT2*-labelled, GFP+ cells (green; arrows) in *Ctnnb1*^iΔPC^ mutant and littermate control (*Pdgfrb(BAC)- CreERT2*^*+/T*^ *Ctnnb1*^*lox/+*^) E14.5 heart sections. GFP+ cells showed reduced migration to the apex and were less abundant in the *Ctnnb1*^iΔPC^ compact myocardium. (**d**) Statistical analysis of *Ctnnb1*^iΔPC^ versus control ventricle size, number of GFP+ cells in E14.5 compact myocardium and relative coverage of ventricle by GFP+ cells (*Ctnnb1*^iΔPC^: *n*=7; control: *n*=8). Error bars±s.e.m. *P* values, two-tailed unpaired *t*-test; NS, no significance.

Next, a potential role of Wnt signalling in mural cell development was tested by combining the *Pdgfrb(BAC)-CreERT2* line and mice carrying loxP-flanked *Frizzled 4* (*Fzd4*^*lox/lox*^) or β-catenin (*Ctnnb1*^*lox/lox*^) alleles in the *Rosa26-mTmG* Cre reporter background. The resulting *Fzd4*^iΔPC^ and *Ctnnb1*^iΔPC^ mice were used to investigate the role of Wnt signalling in PDGFRβ+ cells during their development from mesenchymal progenitors to mural cells (Supplementary Table 1). As *Pdgfrb(BAC)-CreERT2* expression is absent in the endothelium, this strategy avoided effects on Wnt signalling in ECs, which is known to control the endothelial–mesenchymal transition of endocardial cells^31^,^32^. Following tamoxifen administration from E9.5 to E12.5, GFP-positive (recombined) cells were abundant in the heart valves and myocardium of control embryos with intact (wild-type) *Fzd4* alleles at E14.5 (Supplementary Fig. 10a–f). Analysis of homozygous *Fzd4*^iΔPC^ (*Pdgfrb(BAC)-CreERT2 Fzd4*^*lox/lox*^) mutants generated with the same experimental strategy revealed that GFP+ cells in the compact myocardium were significantly reduced relative to *Fzd4* heterozygous (*Pdgfrb(BAC)-CreERT2 Fzd4*^*lox/+*^) littermate controls, whereas ventricle size, width and area were comparable in both groups (Fig. 6a,b and Supplementary Fig. 10g,h). As this experimental strategy only led to rare GFP labelling of cells in and near the epicardium, the reduction of *Fzd4*^iΔPC^ GFP+ cells in the myocardium and towards the cardiac apex is likely to reflect defective migration of progenitors from the AVC and OFT (Fig. 6a,b). To address whether canonical, β-catenin-mediated Wnt signalling^33^ is required for mural cell development, the same tamoxifen-inducible approach was used to generate *Ctnnb1*^iΔPC^ (*Pdgfrb(BAC)-CreERT2 Ctnnb1*^*lox/lox*^) mutants and *Pdgfrb(BAC)-CreERT2 Ctnnb1*^*lox/+*^ heterozygous controls in the *Rosa26-mTmG* background. After tamoxifen administration, the abundance of GFP+ cells was significantly reduced in the E14.5 *Ctnnb1*^iΔPC^ compact myocardium (Fig. 6c,d). In contrast, ventricle size, width and area were not significantly altered in *Ctnnb1*^iΔPC^ mutants indicating the defects were not induced by developmental delay (Fig. 6d; Supplementary Fig. 10i). Altogether, these data argue that the migration of PDGFRβ+ precursors from the AVC/OFT requires Frizzled 4 and canonical Wnt signalling in a cell-autonomous fashion. While the pattern of GFP expression under control of the *Rosa26-mTmG* Cre reporter indicates that our tamoxifen administration strategy avoided appreciable recombination in epicardial cells (Supplementary Fig. 10a), the existence of other potential sites of PDGFRβ+ precursors outside the AVC/OFT cannot be ruled out based on the genetic experiments presented above. However, prolonged tamoxifen administration from E9.5 to E12.5, which might have targeted such cells, was necessary to achieve sufficient *Pdgfrb(BAC)-CreERT2*-mediated recombination in midgestation embryos.

**Figure 7:**
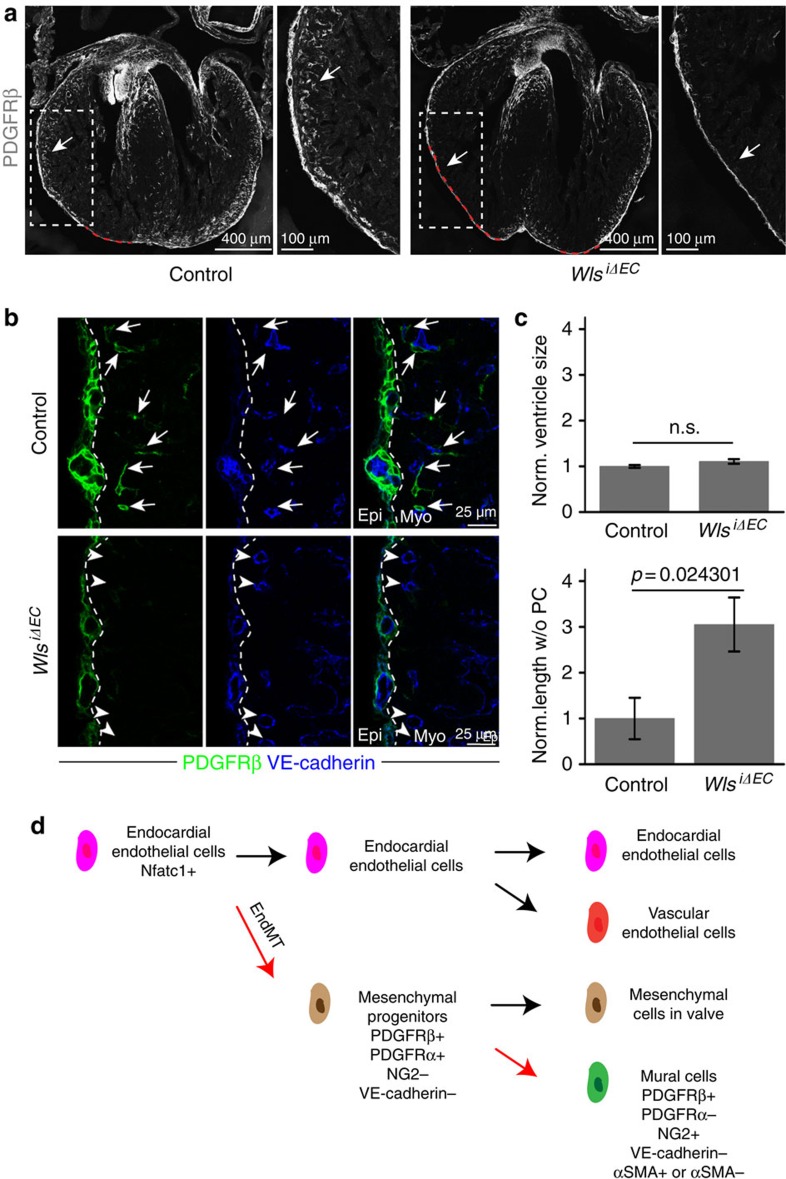
EC-derived Wnt ligands are necessary for mural cell recruitment in compact myocardium. (**a**) Maximum intensity projections showing PDGFRβ+ cells in E14.5 *Wls*^iΔEC^ (right) and littermate control (*Wls*^*lox/lox*^) heart sections and higher magnifications (insets) of compact myocardium. Red dashed lines in overview images mark regions of compact myocardium without PDGFRβ+ cells, which were used for quantitation shown in **c**. (**b**) Representative single plane images showing low PDGFRβ+ (green) cell coverage of VE-cadherin immunostained (blue) blood vessels in *Wls*^iΔEC^ compact myocardium relative to control. Arrows mark perivascular PDGFRβ+ cells, arrowheads indicate blood vessels without PDGFRβ+ coverage. PDGFRβ+ cells were abundant in *Wls*^iΔEC^ and control epicardium (left of dashed lines). Myo, myocardium; Epi, epicardium. (**c**) Statistical analysis of *Wls*^iΔEC^ ventricle size and length of region in compact myocardium devoid of PDGFRβ+ cells (see red dashed lines in **a**) normalized to control (*n*=5 per group). Error bars±s.e.m. *P* values, two-tailed unpaired *t*-test; NS, no significance. (**d**) Endocardial cell lineage tree in the heart. Nfatc1+ embryonic endocardial endothelial cells undergo endothelial–mesenchymal transition (EndMT) and differentiate to primitive (PDGFRβ+ PDGFRα+ NG2− VE-cadherin−) mesenchymal progenitors in AVC/OFT. These mesenchymal progenitors proliferate and populate heart valves but also migrate to the myocardium and differentiate into cardiac mural cells (PDGFRβ+ PDGFRα− NG2+ VE-cadherin- αSMA+/αSMA−).

The transmembrane protein Wls/Evi is essential for Wnt ligand secretion^34^, which allowed us to explore whether the recruitment of PDGFRβ+ mural cells during heart development might be controlled by EC-derived signals. Tamoxifen-inducible *Cdh5(PAC)-CreERT2* transgenic animals were bred to conditional *Wls*^tm1.1Lan^ (*Wls*^lox/lox^) mice to generate EC-specific *Wls*^iΔEC^ mutants. Tamoxifen administration from E11.5 to E12.5 led to profound defects in mural cell recruitment at E14.5 (Fig. 7a–c). In comparison with littermate controls, larger regions in the *Wls*^iΔEC^ compact myocardium were devoid of PDGFRβ+ cells and coronary blood vessels were not properly covered by mural cells (Fig. 7b,c). While this approach might also affect pericyte progenitors in the epicardium, our findings establish that EC-derived Wnt ligands are essential for the recruitment of Fzd4-expressing mural cell progenitors into the developing myocardium.

## Discussion

Pericytes and vSMCs are critical for normal function of the vasculature and, accordingly, changes in these cell population are associated with a number of disease conditions^7^,^18^. Despite the essential roles of these cells, we lack sufficient understanding of their origin during development and turnover in the adult. *Ex vivo* maturation of tumor-derived PDGFRβ+ pericytes involves the upregulation of the markers NG2 and α-smooth muscle actin (αSMA)^35^. However, many fundamental aspects of pericyte biology remain poorly understood, which has several reasons. Due to the tree-like nature of the highly branched vascular network, pericytes are widely scattered within organs. Moreover, there is a lack of strictly pericyte-specific markers, which could, in the absence of morphological criteria such as vessel association, permit the unambiguous isolation and purification of these cells^7^. Likewise, little is known about the identity of pericyte progenitors, their exact location within developing organs and the processes controlling their recruitment and maturation. While early studies have indicated that vessel-associated mural cells are recruited from the surrounding mesenchyme, there is now evidence for numerous distinct developmental sources^7^. Neural crest cells give rise to pericytes in retina, brain, thymus and the head region^36^,^37^,^38^,^39^. In gut, lung and liver, the mesothelium, a single-layer squamous epithelium, is a source of mural cells^7^,^40^,^41^. Likewise, mural cells in the heart are derived from epicardial mesothelial cells^19^,^20^,^21^, which need to undergo epithelial–mesenchymal transition prior to their migration into the myocardium.

Understanding the origin and pathways that drive the development of coronary vasculature is a central question in developmental biology^42^. Our new findings identify endocardial ECs as a novel and unexpected source of cardiac mural cells (Fig. 7d). Thus, the developmental sources of both coronary endothelial cells and pericytes are even more heterogeneous than previously appreciated. While ECs of the outer myocardium are derived from the sinus venosus, the endocardium is the source of the coronary ECs in the inner half of the ventricular wall^25^,^27^,^28^,^43^,^44^. Likewise, coronary mural cells can be either derived from the epicardium or, as we show here, via endothelial–mesenchymal transition from the endocardium. These surprising findings raise the possibility that distinct subsets of vascular cells, depending on their developmental origin, could differentially contribute to pathological processes such as coronary artery disease or fibrotic scarring.

Previous work has established that signalling by β-catenin, Lef1 and TCF is critically required in endocardial cells for endothelial–mesenchymal transition. Heart cushions of EC-specific *Ctnnb1* mutant mice were largely devoid of mesenchymal cells and mutant ECs showed reduced αSMA expression in response to TGFβ2, a known inducer of endothelial–mesenchymal transition^32^. Endocardium-specific inactivation of the gene encoding Tbx20, a T-box family transcription factor, caused embryonic lethality with cushion and valve defects, and reduced expression of Lef1, which was linked to defective Wnt/β-catenin signalling^45^. Defective function of Apc, a negative regulator of β-catenin, led to ectopic endothelial–mesenchymal transition throughout the endocardium of mutant zebrafish^46^. It is important to emphasize that the defective migration and recruitment of *Fzd4*^iΔPC^ or *Ctnnb1*^iΔPC^ mural cell progenitors reported here was not caused by this early role of Wnt/β-catenin signalling. *Pdgfrb(BAC)-CreERT2* transgenic mice show no Cre activity in the endocardium or elsewhere in the cardiac endothelium and GFP reporter-labelled cells were abundant in the mutant AVCs and valves indicating successful endothelial–mesenchymal transition. Thus, our results indicate a distinct role of Fzd4 and β-catenin in mural cell progenitor recruitment to the coronary vasculature. Interestingly, the related receptor Fzd7 was shown to control the polarization and motility of cultured pericytes^47^, which is consistent with our *in vivo* findings.

The relatively small number of reports on EC transdifferentiation *in vivo* suggests that such processes might be rare and confined to a few conditions. During embryonic development, haemogenic ECs in the early aorta give rise to haematopoietic stem cells and thereby initiate definitive hematopoiesis^48^,^49^,^50^,^51^. It also has been reported that cultured ECs can be coerced into the expression of αSMA and other vSMC markers indicative of endothelial–mesenchymal transition^52^,^53^. Genetic approaches suggested conversion of ECs into cells with high expression of smooth muscle actin in mouse models of pulmonary hypertension with neointima formation or during vein graft remodelling^54^,^55^. Conversely, mesenchymal cells can differentiate into ECs contributing to neovascularization during acute ischaemic cardiac injury^56^. It was also reported that activin-like kinase-2 (ALK2) can trigger the differentiation of ECs into multipotent cells that, in turn, give rise to osteoblasts, chondrocytes or adipocytes^57^. Mesenchymal cells generated by endothelial–mesenchymal transition are known to populate heart valves and can promote cardiac fibrosis in response to transforming growth factor β (TGFβ)^29^,^58^. These findings indicate that ECs not only form vascular networks through a series of vessel growth and pruning processes^9^,^59^, but can also have substantial differentiation potential. Our own findings establish that cardiac pericytes and vSMCs are in part derived from the endocardium, which involves a PDGFRβ+ PDGFRα+ NG2− intermediate, which we propose to act as a primitive mesenchymal progenitor. Later in development, this progenitor population gives rise to PDGFRβ+ PDGFRα− NG2+ cells in the myocardium that are tightly associated with capillaries. Interestingly, we also found that heart valves retain PDGFRβ+ PDGFRα+ NG2− cells even after birth. As the presence of these cells could be relevant for pathological processes such as fibrosis or valve calcification, future work should explore the role of PDGFRβ+ PDGFRα+ NG2− cells in cardiac disease and in processes involving endothelial–mesenchymal transition. In this context, it will be important to distinguish between *de novo* endothelial–mesenchmal transitions in the adult from processes involving pre-existing populations of mesenchymal cells that were derived from the endocardium during development.

## Methods

### Generation of *Pdgfrb(BAC)-CreERT2* transgenic mice

A complementary DNA (cDNA) encoding tamoxifen-inducible Cre recombinase (CreERT2) followed by a polyadenylation signal sequence and an FRT-flanked ampicillin resistance cassette was introduced by recombineering into the start codon of *Pdgfrb* in the BAC clone RP24-62H17TJ (BACPAC Resources Center, Children's Hospital Oakland Research Institute, Oakland, USA). After Flp-mediated excision of the ampicillin resistance cassette in bacteria, the resulting constructs were validated by PCR analysis and used in circular form for pronuclear injection into fertilized mouse oocytes. Founders, identified by PCR genotyping, were screened by timed matings with *Rosa26-mTmG* Cre reporter animals^60^. Cre activity was induced by three consecutive intraperitoneal injections of pups at postnatal (P) days 1–3 with 50 μg tamoxifen solution (Sigma, T5648; 10 mg ml^−1^; generated by diluting a 10^3^ tamoxifen stock in 100% ethanol with peanut oil). Faithful recombination in embryonic, postnatal and adult PDGFRβ+ cells was validated by combining *Pdgfrb(BAC)-CreERT2*-mediated GFP expression and anti-PDGFRβ immunostaining.

### Genetically modified mice and inducible genetic experiments

Following overnight mating, female mice were examined in the following morning for the presence of a vaginal plug, which was counted as embryonic day 0.5 (E0.5). C57BL/6 mice were used for all analysis using wild-type hearts.

The mouse models used in this study are summarized in Supplementary Table 1. *Pdgfra*^*tm11(EGFP)Sor*^ (ref. 61) and *Tg(Cspg4-dsRed.T1)*^*1Akik*^_(ref. 62) mice were used as reporter mice for PDGFRα and NG2 cells, respectively. In the *Rosa26-mTmG* reporter^60^, Cre activity leads to an irreversible switch from constitutive expression of membrane-anchored tdTomato protein to membrane-anchored GFP. *Rosa26-mTmG* Cre reporter animals were interbred with *Pdgfrb(BAC)-CreERT2* transgenic mice for clonal analysis or functional studies in PDGFRβ+ cells. For genetic cell fate tracking, 1 mg 4-hydroxytamoxifen (4-OHT; Sigma, H7904) was injected intraperitoneally at E10.5 into pregnant females for analysis at later time points. In gene inactivation experiments, 3 mg tamoxifen (Sigma, T5648; 15 mg ml^−1^; generated by diluting a 10^3^ tamoxifen stock in 100% ethanol with peanut oil) were injected intraperitoneally to pregnant females once a day from E9.5 to E12.5. For studies in postnatal mice, 50 μg tamoxifen per day were injected from P1 to P3 followed by analysis at P6.

*Rpl22*^*tm1.1Psam*^ (ref. 30) mice were interbred with the *Pdgfrb(BAC)-CreERT2* transgenic line for RiboTag quantitative PCR with reverse transcription (RT-qPCR) analysis. Following daily intraperitoneal injections of 3 mg tamoxifen from E9.5 to E12.5, pregnant females were killed and embryos isolated at E13.5.

For loss-of-function genetics, *Fzd4*^tm2.1Nat^ (ref. 63) or *Ctnnb1*^tm2Kem^ (ref. 64) conditional mutants were interbred with *Pdgfrb(BAC)-CreERT2 Rosa26-mTmG* double-transgenic mice. Gene inactivation was triggered by daily intraperitoneal injections of 3 mg tamoxifen from E9.5 to E12.5. *Pdgfrb(BAC)-CreERT2*^*+/T*^ *Fzd4*^*lox/+*^ (*n*=8) *or Pdgfrb(BAC)-CreERT2*^*+/T*^ *Ctnnb1*^*lox/+*^ (*n*=8) embryos in the *Rosa26-mTmG* Cre reporter background were used as controls for *Pdgfrb(BAC)-CreERT2*^*+/T*^ *Fzd4*^*lox/lox*^ (*Fzd4*^iΔPC^, *n*=8) and *Pdgfrb(BAC)-CreERT2*^*+/T*^ *Ctnnb1*^*lox/lox*^ (*Ctnnb1*^iΔPC^, *n*=7) mutants, respectively.

*Wls*^tm1.1Lan^ (ref. 65) conditional mutants were interbred with the *Cdh5(PAC)-CreERT2* (ref. 66) transgenic line to generate *Cdh5(PAC)-CreERT2*^*+/+*^ *Wls*^*lox/lox*^(control, *n*=5) and *Cdh5(PAC)-CreERT2*^*+/T*^ *Wls*^*lox/lox*^ (*Wls*^iΔEC^, *n*=5) embryos. Pregnant females were intraperitoneally injected with tamoxifen (2 mg) at E11.5 and E12.5 before analysis at E14.5. *Tg(BAT-lacZ)*^3Picc^ (ref. 67) mice were isolated at E13.5 for analysis of Wnt activation. *Apln-CreERT2* (ref. 26), *Cdh5(PAC)-CreERT2* (ref. 66), *Tie2-Cre*^68^ and *Wnt1-Cre*^69^ were interbred with *Rosa26-mTmG* reporter mice for tracking of endothelial cells or neural crest lineage, respectively.

*Nfatc1-CreERT2* (ref. 25) mice were combined with *Rosa26-RFP*^70^ reporters for analysis of endocardial cell-derived cell populations. In these experiments, pregnant females received 6 mg 4-OHT at E8.5.

All animal experiments were performed in compliance with the relevant laws and institutional guidelines and were approved by local animal ethics committees.

### Cryosectioning and immunohistochemistry

At E9.5 and E10.5, isolated whole embryos were immediately placed in ice-cold 4% paraformaldehyde (PFA-PBS) solution and fixed under gentle agitation overnight. At E14.5 or E16.5, hearts were dissected prior to overnight fixation in PFA-PBS. Next, samples were dehydrated in 20% sucrose-PBS solution overnight, after which E10.5 hearts were dissected. Hearts were embedded in PBS containing 15% sucrose, 8% gelatin and 1% polyvinylpyrrolidone for storage at −80 °C and cryosectioning on a Leica CM3050 cryostat using low profile blades. Typically, 30 μm sections were cut in transverse (ventral to dorsal) orientation for immunohistochemistry unless stated otherwise. In *Pdgfrb(BAC)-CreERT2 Rosa26-mTmG* clonal analysis experiments, <5% of E10.5 heart slices were missed.

For immunostaining, heart sections were rehydrated in PBS, permeabilized for 15 min in 0.5% Triton X100 PBS and blocked for 30 min in PBS containing 1% BSA, 2% donkey serum, 0.3% Trion X100 PBS (blocking buffer) at room temperature. Sections were probed with primary antibodies diluted in blocking buffer at 4 °C overnight. After incubation, sections were washed three times with PBS and incubated with appropriate Alexa Fluor-conjugated secondary antibodies (1:100 to 1:400, Invitrogen) diluted in blocking buffer at room temperature for 2 h. Nuclei were stained with Hoechst or DAPI during secondary antibody incubation. After that, sections were washed three times with PBS, mounted with Fluoromount-G (0100-01,Southern Biotech) and kept in 4 °C for imaging.

The following primary antibodies were used: PECAM1 (553370, Pharmingen, 1:100), PDGFRβ (14-1402-82, eBioscience, 1:100), PDGFRα (14-1401-81, eBioscience, 1:100), isolectin B4 (B-1205, Vector, 1:100), NG2 (AB5320, Millipore, 1:100), PDGFRα (3164, Cell Signaling, 1:100), GFP (A21311, Invitrogen, 1:100), GFP (GFP-1010, Aves, 1:200), VE-cadherin (AF1002, R&D Systems, 1:100), CD13 (MCA2183GA, AbD Serotec, 1:100), Collagen IV (2150-1470, AbD Serotec, 1:100), Desmin (ab15200, Abcam, 1:100), Tbx18 (sc-17869, Santa Cruz, 1:100), αSMA (eBioscience, 50-9760-82, 1:100), oestrogen receptor (ab27595, Abcam, not diluted), β-galactosidase (ab9361, Abcam, 1:100), RFP (600-401-379, Rockland, 1:1,000), and RFP (ABIN334653, ChromoTEK, 1:200). For the staining of oestrogen receptor, prediluted primary antibody was directly added to sections at 4 °C overnight. A goat-anti rabbit HRP (1:500, G21234, Invitrogen) secondary antibody was incubated for 1 h. The TSA Plus Cyanine 3 System Kit (1:70, NEL744001KT, Perkin Elmer) was finally used for 5 min and sections were immediately washed in PBS after exposure. For clonal analysis in *Nfatc1-CreERT2 Rosa26-RFP* mice, PDGFRβ/NG2/PDGFRα signals at E10.5 and PDGFRβ/NG2 signals at E16.5 were generated with the tyramide signal amplification kit (Life Technologies).

### Flow cytometry and cell culture

*Tie2-Cre Rosa26-mTmG* double-transgenic embryos were killed at indicated developmental stage. Hearts were dissected and manually cut into top and bottom parts for analysis. 4 to 5 heart fragments were pooled and minced with a sterilized razor blade. The tissue was immersed in 0.5 ml dissociation solution (20% FCS-PBS solution with 145 U ml^−1^ type 2 and type 4 Gibco collagenase) and incubated at 37 °C for 60 min. The samples were passed through a 19 G needle for several times and filtered using 40 μm Nylon cell strainer. The remaining tissue was treated with red blood cell lysing solution on ice for 10 min and blocked using 2% FCS-PBS solution on ice for 30 min. Primary antibody was incubated for 60 min on ice and diluted in 2% FCS-PBS solution. PDGFRβ-APC (1:50, 17-1402-82, eBioscience) was used to detect PDGFRβ cells while a rat IgG2a-APC antibody was used as isotype control (1:50, 17-4321, eBioscience). Cells were resuspended in PBS/2%FCS supplemented with 1 μg ml^−1^ DAPI to allow exclusion of nonviable cells. Cell sorting was performed on a FACSAria IIu cell sorter (BD Biosciences, San Jose, CA) using a 85 μm nozzle.

FACS-sorted cells were cultured in 24- or 48-well dishes coated with 0.2% gelatin (G1393, Sigma). Cells were initially cultured in Endothelial Cell Growth Medium 2 (EGM2) (CC-3156 and CC-4176, Lonza). To analyse the differentiation potential of these cells, EGM2 culture medium was replaced with StemXVivo Osteogenic/Adipogenic Base Media (R&D, CCM007) supplemented with Osteogenic Supplement (R&D, CCM009) or Adipogenic Supplement (R&D, CCM011) when cells had reached 50 to 70% confluency. Adipocytes and osteocytes were detected using Oilred O staining (O0625, Sigma) and Alizarin Red staining (TMS-008-C, Millipore) after culture for 21 days, respectively. Alternatively, EGM2 was replaced by pericyte culture medium (ScienCell, #1201) supplemented with 2%FBS (ScienCell, #0010), pericyte growth supplement (ScienCell, #1252) and penicillin/Streptomycin after 7 days of culture. After further 7 days in pericyte medium, these cells were fixed by 4% PFA-PBS for immunostaining. Cell morphology was documented with a Zeiss AxioObserver.

### Confocal imaging and image processing

Stained heart sections were imaged with laser scanning confocal microscopes (Leica SP5 or SP8, Zeiss LSM510, and Olympus FV1000) after immunohistochemistry. Quantitative analysis of mutant phenotypes was done with a Leica SP5 microscope and identical imaging acquisition setting for mutant and control samples. Overview images of hearts were automatically scanned using the tile-scan function of Leica confocal microscope. Volocity (PerkinElmer), Fiji (open source; http://fiji.sc/), Photoshop and Illustrator (Adobe) softwares were used for image processing in compliance with requirements of *Nature Communications*. In general, original images were loaded into Volocity. Brightness-contrast modification was applied to the whole image. Images exported from Volocity were rotated and cropped in Fiji. Quantification of cell number, length and area was performed in Volocity and Fiji.

To quantify ventricle areas, we used the following steps to process original tdTomato images from *Pdgfrb(BAC)-CreERT2 Rosa26-mTmG* hearts. First, tdTomato signal in atria was removed using the ‘polygon selections and fill' function in Fiji. Next, the ventricle image was loaded into Photoshop, contrast/brightness were modified and ventricles were segmented using ‘magic wand tool'. Finally, area of ventricle was selected and calculated in Volocity with the ‘find objects using intensity' function excluding objects smaller than 500 μm^2^.

The size of a ventricle was defined as the total ventricle area including the lumen (indicated by the white dashed line in Supplementary Fig. 10a). GFP cell coverage was defined as the ventricle area containing GFP+ cells (cyan dashed line in Supplementary Fig. 10a). The normalized length of compact myocardium without PDGFRβ+ cells (PC; Fig. 7c) refers to the relative length indicated by the red dashed line in the representative overview images (Fig. 7a) normalized to control.

### RiboTag and quantitative PCR analysis

For gene expression analysis in cardiac PDGFRβ+ cells, the RiboTag method was used to enrich ribosome-associated transcripts. In brief, hearts from *Pdgfrb(BAC)-CreERT2 Rpl22*^*tm1.1Psam*^ double-transgenic mice were freshly dissected and immediately transferred into liquid nitrogen. Next, samples were immersed in polysome buffer (50 mM Tris pH7.5, 100 mM KCl, 12 mM MgCl_2_, 1mM DTT, 200 U ml^−1^ RNase inhibitor, Protease inhibitor cocktail (P2714, Sigma), 1 mg ml^−1^ heparin, 100 μg ml^−1^ cycloheximide, 1% IGEPAL CA-630) and homogenized with pellet pestles. Samples were centrifuged for 10 min with 16,000*g* to remove debris. 2.5% of supernatant was kept as ‘input' for direct RNA extraction. Anti-HA antibody (M180-9, MBL) conjugated with magnetic beads, which were pre-washed in immunoprecipitation buffer (50 mM Tris pH7.5, 100 mM KCl, 12 mM MgCl_2_, 1% IGEPAL CA-630), was added to the remaining supernatant and incubated under rotation at 4 °C overnight. Following the aspiration of supernatant, beads were washed with high salt buffer (50 mM Tris pH7.5, 300 mM KCl, 12 mM MgCl_2_, 1 mM DTT, 100 μg ml^−1^ cycloheximide, 1% IGEPAL CA-630) for 4 times. Supernatant was completely aspirated after the final wash and beads were used for RNA extraction as ‘HA enrichment'.

RNAs from both ‘input' and ‘HA enrichment' were extracted using RNeasy plus Micro Kit (74034, Qiagen). cDNA were generated with iScript cDNA Synthesis kit (#170-8891, BioRad). Quantitative PCR with reverse transcription was performed by ABI Prism 7900HT fast real-time PCR system using Taqman probes. The Taqman probes were mixed with Taqman gene expression master mix (4369016, Life technogies). The FAM-conjugated Taqman probes included *Pdgfrb* (Mm00435546_m1), *Cdh5* (Mm00486938_m1), *Pecam1* (Mm01242584_m1), *Ctnnb1* (Mm00483039_m1), *Fzd4* (Mm00433382_m1), *Lrp5* (Mm01227476_m1), *Lrp6* (Mm00999795_m1), *Lef1* (Mm01310389_m1), *Tcf4* (Mm00443210_m1), *Axin2* (Mm01265780_m1). Gene expression levels were normalized to the endogenous VIC-conjugated *Gapdh* probe (4352339E) as control. RNAs from ‘HA enrichment' group were compared with ‘input' group to detect enrichment levels of genes in PDGFRβ+ cells. Threefold increases were considered to be significant. qPCR experiments were repeated three times. To collect enough RNA for qPCR, 4 E13.5 hearts from *Pdgfrb(BAC)-CreERT2 Rpl22* mice were combined for each experiment.

### Statistics

No statistical methods were used to predetermine sample size. The developmental stage of embryos was determined based on vaginal plug check dates and a standard development atlas. Dead embryos and visibly abnormal embryos were excluded from analysis, which was a pre-established criterion before the experiment. No randomization and blinding were used. Samples were tested using two-tailed Student's *t*-test. *P* value <0.05 was considered to be statistically significant. Statistical data were drawn from normally distributed group. Before the Student's *t*-test, samples from different groups were tested using F-test to identify the variances between groups. F value <0.05 indicated samples have significantly different variances. All results are represented as mean±s.e.m. Number of animals or cells represents biological replicates.

### Data availability

Data supporting the findings of this study are available within the article and its Supplementary Information files and from the corresponding author on reasonable request.

## Additional information

**How to cite this article:** Chen, Q. *et al*. Endothelial cells are progenitors of cardiac pericytes and vascular smooth muscle cells. *Nat. Commun.* 7:12422 doi: 10.1038/ncomms12422 (2016).

## Supplementary Material

Supplementary Information Supplementary Figures 1-10, Supplementary Table 1
