# Supplementary material for: Endothelial cells are progenitors of cardiac pericytes and vascular smooth muscle cells
Source: Nat Commun. 2016 Aug 12;7:12422. doi: 10.1038/ncomms12422 (PMC4990645; doi:10.1038/ncomms12422)
Supplement: Supplementary Information — Supplementary Figures 1-10, Supplementary Table 1 [file ncomms12422-s1.pdf]

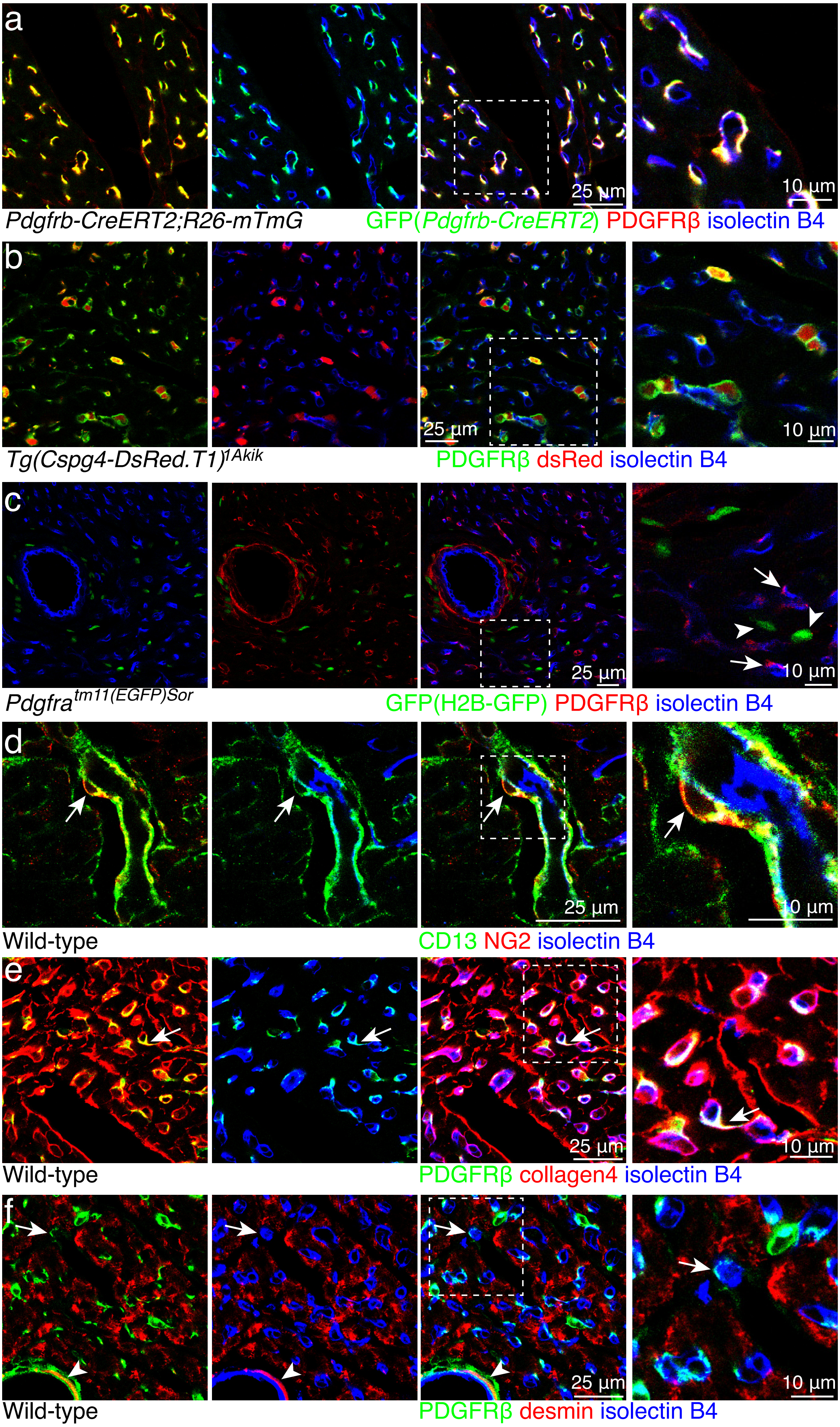

a
25 µm
10 µm
Pdgfrb-CreERT2;R26-mTmG
GFP(Pdgfrb-CreERT2) PDGFRβ isolectin B4
b
25 µm
10 µm
Tg(Cspg4-DsRed.T1)1Akik
PDGFRβ dsRed isolectin B4
c
25 µm
10 µm
Pdgfratm11(EGFP)Sor
GFP(H2B-GFP) PDGFRβ isolectin B4
d
25 µm
10 µm
Wild-type
CD13 NG2 isolectin B4
e
25 µm
10 µm
Wild-type
PDGFRβ collagen4 isolectin B4
f
25 µm
10 µm
Wild-type
PDGFRβ desmin isolectin B4

Supplementary Figure 1 l Molecular markers for capillary-associated cardiac mural cells at postnatal heart.
(**a**) Genetic labeling of mural cells in the P6 heart. Following *Pdgfrb(BAC)-CreERT2*-mediated recombination of the *Rosa26-mTmG* Cre reporter, GFP expression (green) overlapped with PDGFRβ immunostaining (red) around myo-cardial capillary ECs (isolectin B4, blue).
(**b**) *Tg(Cspg4-dsRed.T1)*^{1Akik} reporter expression (dsRed, red) indicates overlap-ping expression of NG2/Cspg4 and PDGFRβ (green) in vessel-associated (isolectin B4, blue) cells.
(**c**) *Pdgfra*$^{tm11(EGFP)Sor}$ (nuclear H2B-GFP, green) labeling of PDGFRα+ interstitial cells (arrowheads) but not of PDGFRβ+ (red) vessel-associated (isolectin B4, blue) mural cells (arrows) at P6.
(**d**-**f**), Confocal images of P6 wild-type myocardium after staining for the indicated markers. Arrows mark cardiac mural cells associated with capillaries (isolectin B4, blue). Desmin (red, **f**) staining is abundant in cardiomyocytes.
Images on the right show higher magnifications of corresponding insets.

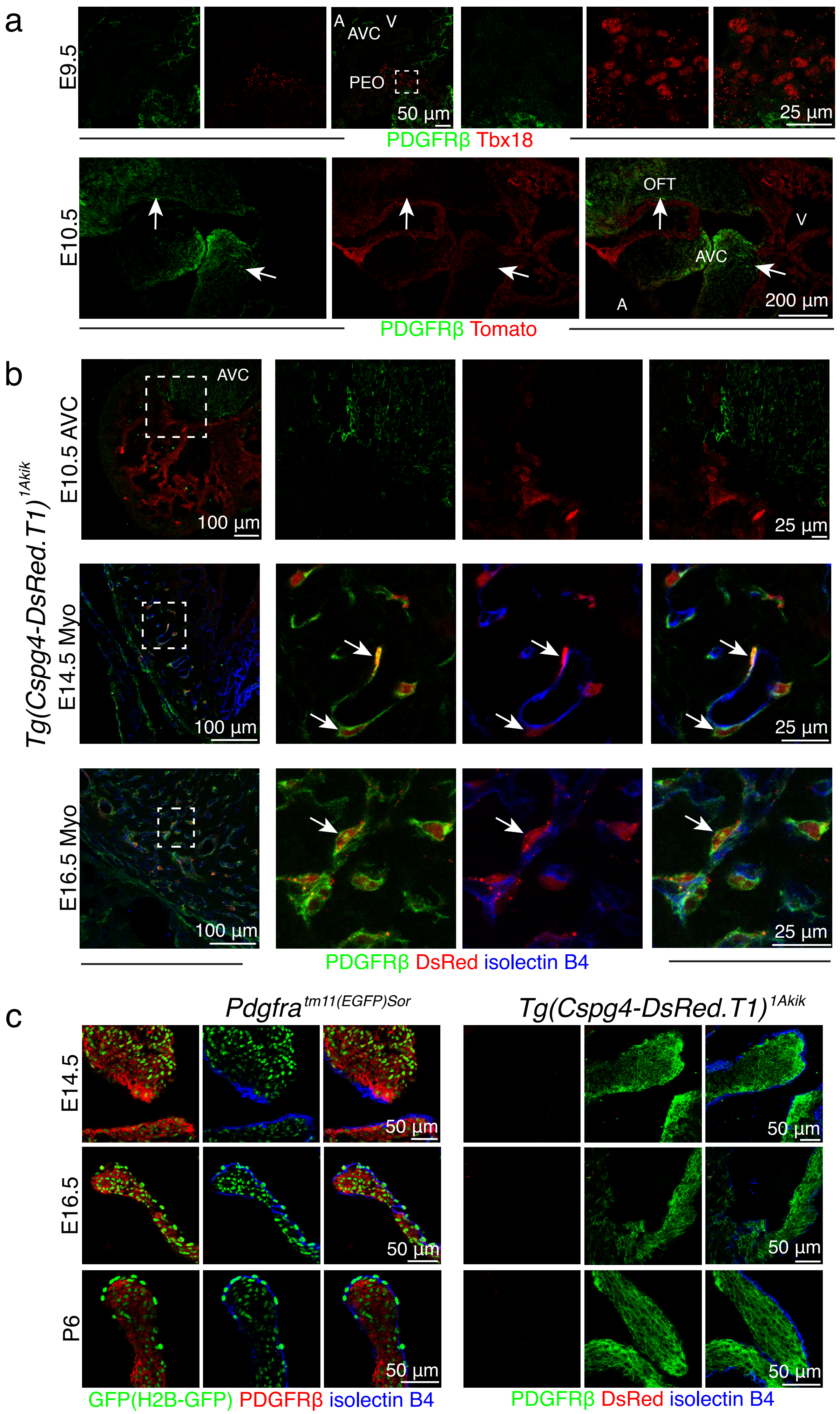
a
E9.5
A
AVC
V
PEO
50 µm
25 µm
PDGFRβ Tbx18
E10.5
OFT
V
AVC
A
200 µm
PDGFRβ Tomato
b
Tg(Cspg4-DsRed.T1)1Akik
E10.5 AVC
AVC
100 µm
25 µm
E14.5 Myo
100 µm
25 µm
E16.5 Myo
100 µm
25 µm
PDGFRβ DsRed isolectin B4
c
Pdgfratm11(EGFP)Sor
Tg(Cspg4-DsRed.T1)1Akik
E14.5
E16.5
P6
50 µm
GFP(H2B-GFP) PDGFRβ isolectin B4
PDGFRβ DsRed isolectin B4

Supplementary Figure 2 I Distribution and molecular properties of PDGFRβ cells in heart.

(**a**) PDGFRβ protein (green) expression pattern in the E9.5 and E10.5 heart, as indicated. At E9.5, PDGFRβ+ cells were found around the atrioventricular canal (AVC) but were absent from the Tbx18+ (red) proepicardial organ (PEO). Panels on the right show higher magnification of inset. PDGFRβ-immunostained cell clusters (arrows in bottom row) were located in the outflow tract (OFT) and the endocardial cushions of the AVC. Heart morphology was visualized by tdTomato protein expression (red) of the unrecombined *Rosa26-mTmG* Cre reporter allele. A, atrium; V, ventricle.

(**b**) *Tg(Cspg4-dsRed.T1)^{1Akik}* expression (DsRed, red) was absent in PDGFRβ (green) immunostained cells in the E10.5 atrioventricular canal (AVC). In contrast, co-expression of both markers was seen in vessel-associated (isolectin B4, blue) cells (arrows) in the E14.5 and E16.5 compact myocardium (Myo).

(**c**) Overlapping PDGFRβ immunostaining (red) and *Pdgfra$^{tm11(EGFP)Sor}$* reporter expression (nuclear H2B-GFP, green) in heart valves at E14.5, E16.5 and P6 (left). In contrast to PDGFRβ (green), *Tg(Cspg4-dsRed.T1)^{1Akik}* expression (DsRed, red) was absent from heart valves at the indicated stages. Endocardial ECs, isolectin B4 (blue).

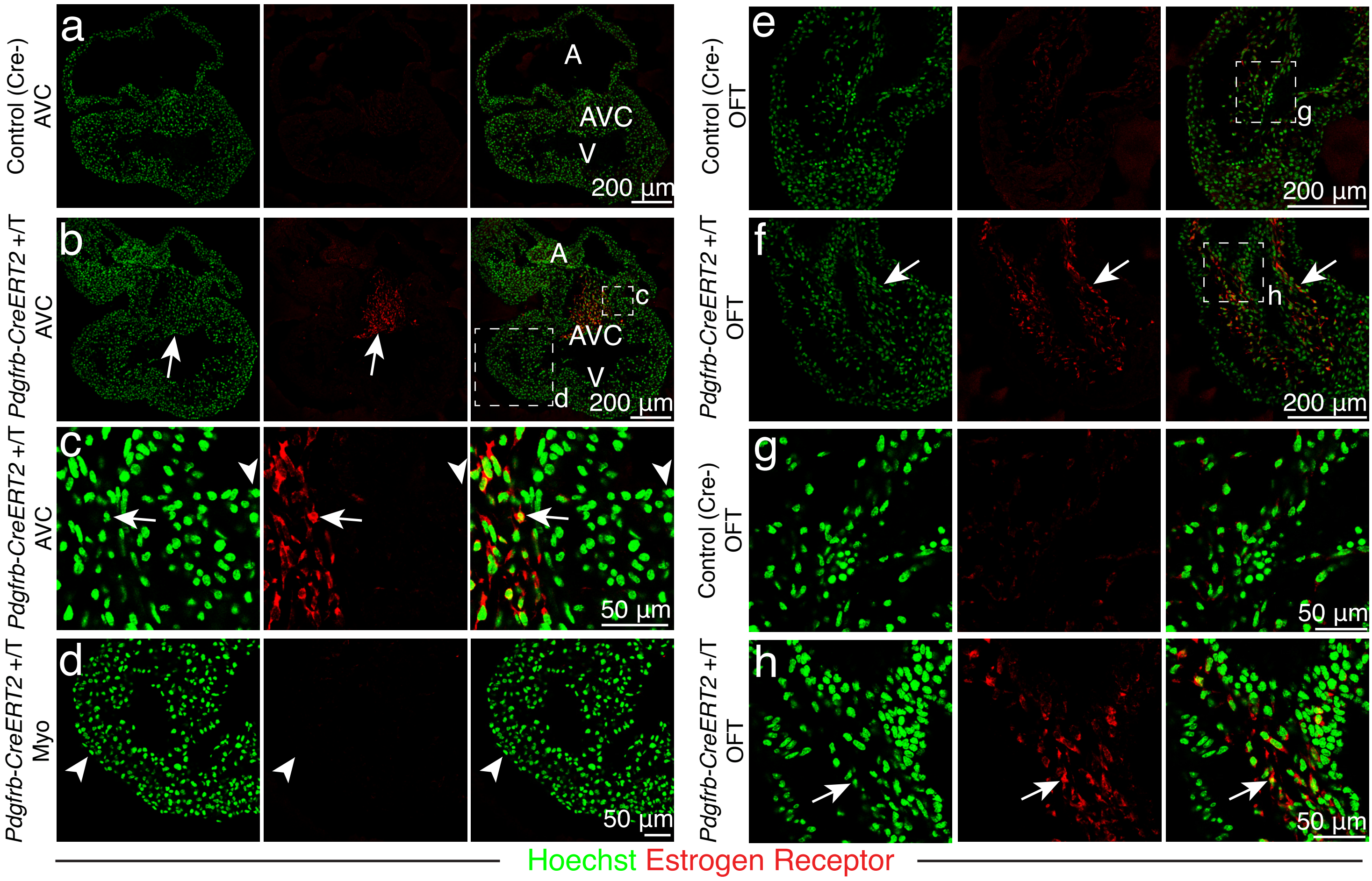

Supplementary Figure 3 I Verification of *Pdgfrb(BAC)-CreERT2* activity in heart.
(**a**-**h**) Immunostaining of CreERT2 (estrogen receptor, red) in E10.5 *Pdgfrb(BAC)-CreERT2* transgenic and Cre-negative littermate control hearts, as indicated. Overview pictures show specific labeling of CreERT2+ cells (arrows) in the E10.5 atrioventricular canal (AVC) (**b**). High magnification of insets in (**b**) shows CreERT2 expression in cells of the AVC (**c**) but not in the E10.5 epicardium (arrowheads) or myocardium (Myo) (**d**). CreERT2+ staining (arrows) was also seen in cells of the E10.5 outflow tract (OFT) (**f** and **h**) but was absent in Cre-negative control sections (**e** and **g**).

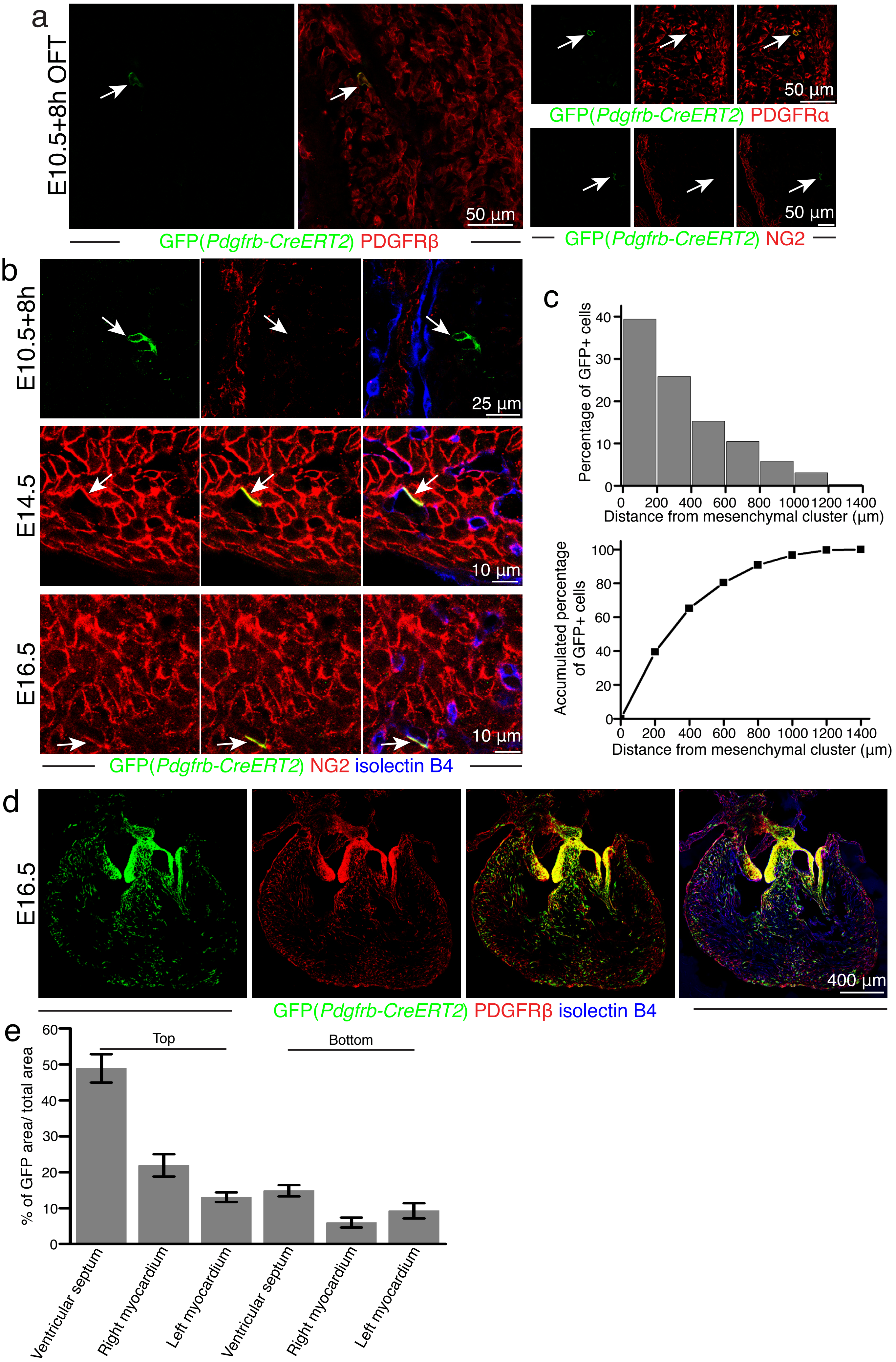

a
E10.5+8h OFT
50 µm
GFP(Pdgfrb-CreERT2) PDGFRβ
50 µm
GFP(Pdgfrb-CreERT2) PDGFRα
50 µm
GFP(Pdgfrb-CreERT2) NG2
b
E10.5+8h
25 µm
E14.5
10 µm
E16.5
10 µm
GFP(Pdgfrb-CreERT2) NG2 isolectin B4
c
Percentage of GFP+ cells
0
10
20
30
40
0 200 400 600 800 1000 1200 1400
Distance from mesenchymal cluster (µm)
Accumulated percentage of GFP+ cells
0
20
40
60
80
100
0 200 400 600 800 1000 1200 1400
Distance from mesenchymal cluster (µm)
d
E16.5
400 µm
GFP(Pdgfrb-CreERT2) PDGFRβ isolectin B4
e
% of GFP area/ total area
0
10
20
30
40
50
60
Top
Bottom
Ventricular septum
Right myocardium
Left myocardium
Ventricular septum
Right myocardium
Left myocardium

Supplementary Figure 4 l Clonal analysis using *Pdgfrb-CreERT2 R26-mTmG* mice in heart.

(**a**) Genetic labeling of cell clones in *Pdgfrb(BAC)-CreERT2 Rosa26-mTmG* double transgenic embryos. 8 hours after 4-hydroxytamoxifen (4-OHT) administration, GFP+ cells (arrows) in the OFT were PDGFRβ+, PDGFRα+ and NG2-.

(**b**) Sections from *Pdgfrb(BAC)-CreERT2 Rosa26-mTmG* double transgenic hearts at the indicated stages after 4-OHT administration at E10.5. Recombined, GFP+ (green, arrows) cell clones were negative for NG2 (red), located in the AVC and not associated with isolectin B4+ (blue) vessels at E10.5+8h. GFP+ NG2+ cells were associated with vessels in the compact myocardium at E14.5 and E16.5.

(**c**) Statistical analysis of the distance of GFP+ cells from the mesenchymal clusters at E14.5 (top) and accumulated distribution of GFP+ cells within the indicated distance range (bottom). Analysis was based on 624 GFP+ cells from 6 E14.5 hearts.

(**d**) Representative distribution of mesenchymal progenitor descendant cells in *Pdgfrb(BAC)-CreERT2 Rosa26-mTmG* double transgenic hearts at E16.5 after consecutive administration of 3mg tamoxifen from E9.5 to E12.5.

(**e**) Analysis of mesenchymal progenitor-derived cell distribution in E16.5 heart based on images as shown in (**d**). The coverage of the indicated regions by GFP+ cells in the top or bottom part of *Pdgfrb(BAC)-CreERT2 Rosa26-mTmG* double transgenic hearts were divided by the total tissue area (excluding lumens). Quantitation was based on 12 sections from 3 E16.5 hearts.

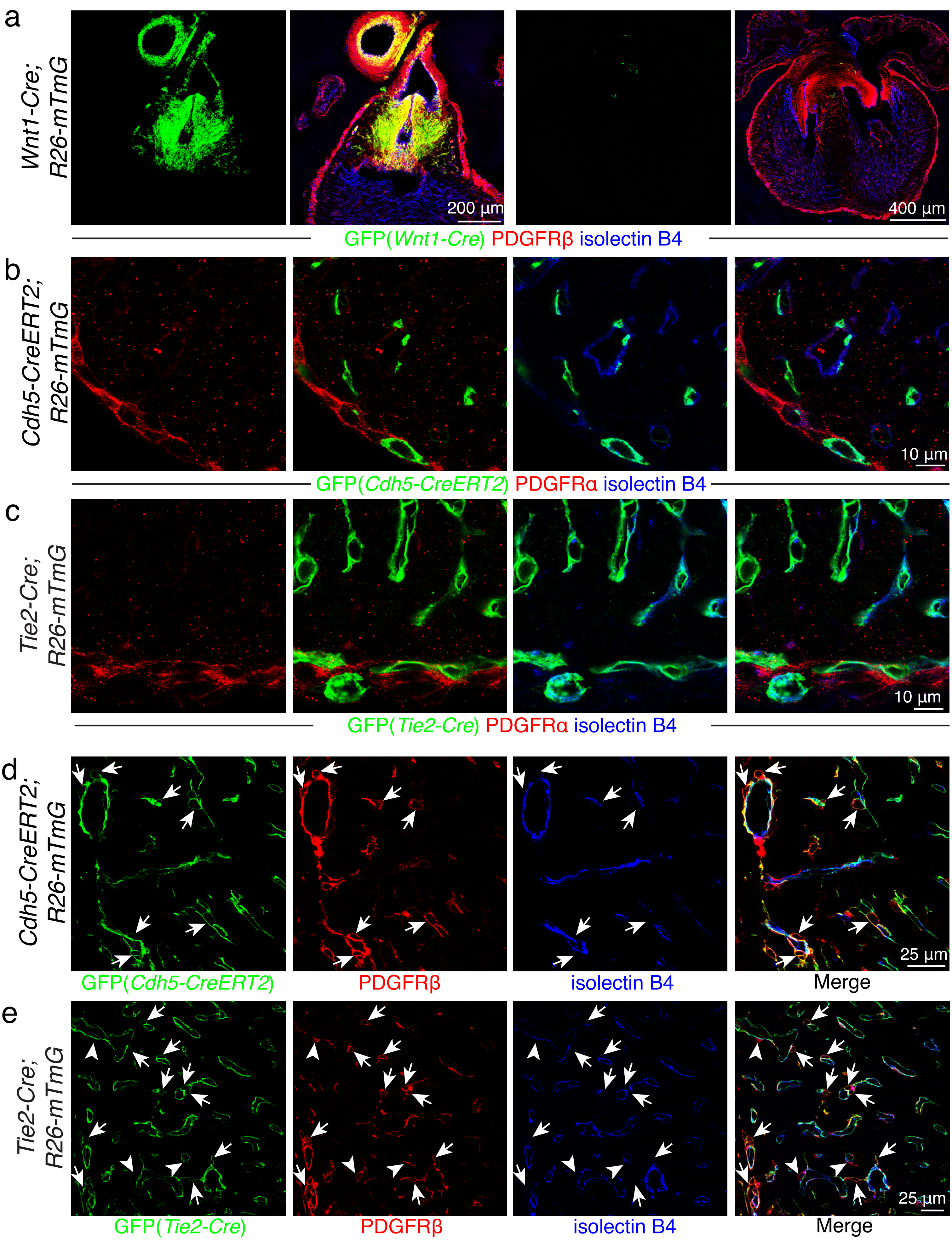

a
Wnt1-Cre; R26-mTmG
200 µm
400 µm
GFP(Wnt1-Cre) PDGFRβ isolectin B4
b
Cdh5-CreERT2; R26-mTmG
10 µm
GFP(Cdh5-CreERT2) PDGFRα isolectin B4
c
Tie2-Cre; R26-mTmG
10 µm
GFP(Tie2-Cre) PDGFRα isolectin B4
d
Cdh5-CreERT2; R26-mTmG
25 µm
GFP(Cdh5-CreERT2)
PDGFRβ
isolectin B4
Merge
e
Tie2-Cre; R26-mTmG
25 µm
GFP(Tie2-Cre)
PDGFRβ
isolectin B4
Merge

Supplementary Figure 5 l Lineage tracing of neural crest and endothelial cells.
(**a**) Analysis of *Wnt1-Cre*-mediated recombination in the *Rosa26-mTmG* reporter background at E14.5. Note abundant GFP staining in the pulmonary valves (left panels) and other structures, but sparse signal in the heart ventricle (right panels).
(**b**) Clonal analysis in *Cdh5-CreERT2 Rosa26-mTmG* double transgenic hearts at E16.5 following 4-OHT administration at E8.5. Higher magnification images show PDGFRα expression in epicardium but not in GFP+ cells in the myocardium.
(**c**) Lineage tracing in *Tie2-Cre Rosa26-mTmG* double transgenic hearts at E16.5. Higher magnification images show PDGFRα signal in the epicardium but not in GFP+ cells in myocardium.
(**d**) Clonal analysis in *Cdh5-CreERT2 Rosa26-mTmG* double transgenic hearts at E16.5 following 4-OHT administration at E8.5. Note abundance of EC-derived GFP+ PDGFRβ+ mural cells (arrows). Images shown are separated channels of Fig. 3c.
(**e**) Lineage tracing in *Tie2-Cre Rosa26-mTmG* double transgenic hearts at E16.5. EC-derived GFP+ PDGFRβ+ mural cells (arrows) were highly abundant. Arrowheads indicate mural cells without GFP labeling and therefore of different developmental origin.

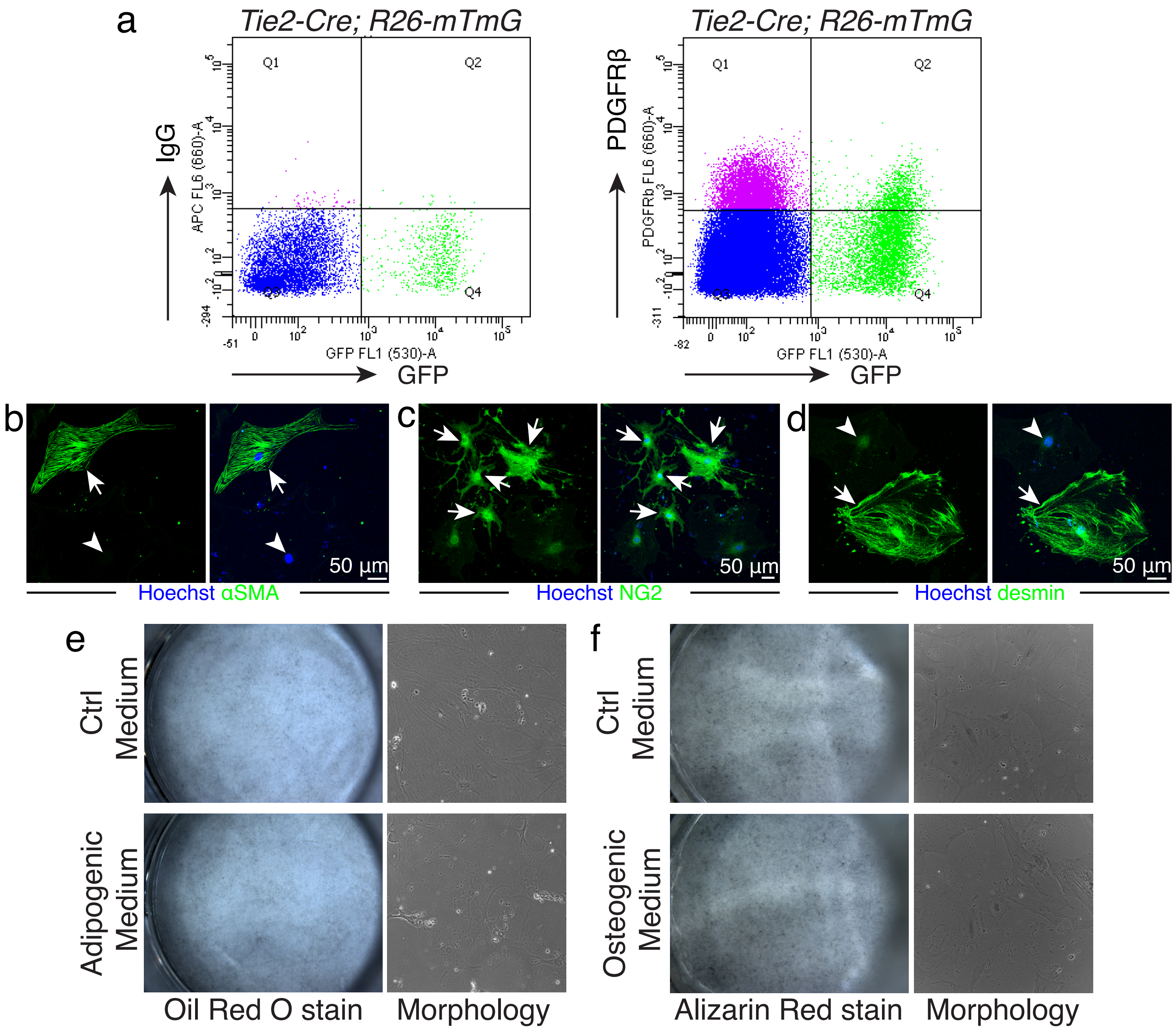

Supplementary Figure 6 I Differentiation potential of EC-derived mesenchymal progenitors
(**a**) Representative FACS plots from E12.5 *Tie2-Cre Rosa26-mTmG* heart. Cells in left panel were stained with isotype control antibody. EC-derived GFP+ PDGFRβ+ cells in Q2 of right panel were isolated for culture.
(**b-d**) Cell differentiation results in pericyte culture medium. Arrows indicate positive cells and arrow-heads negative cells after staining for αSMA (**b**), NG2 (**c**) and desmin (**d**).
(**e**) Cell differentiation in adipogenic medium. Base medium was used as control, while base medium with adipogenic supplement was used to induce adipocyte formation. Left panels indicate absence of Oil Red O staining, right panels show morphology of cells after 21 days in culture.
(**f**) Cell differentiation in osteogenic medium. Base media was used as control, whereas osteogenic supplement was added to induce osteocyte formation. Left panels show absence of Alizarin Red staining in cell pellets. Right panels show typical cell morphology after 21 days in culture.

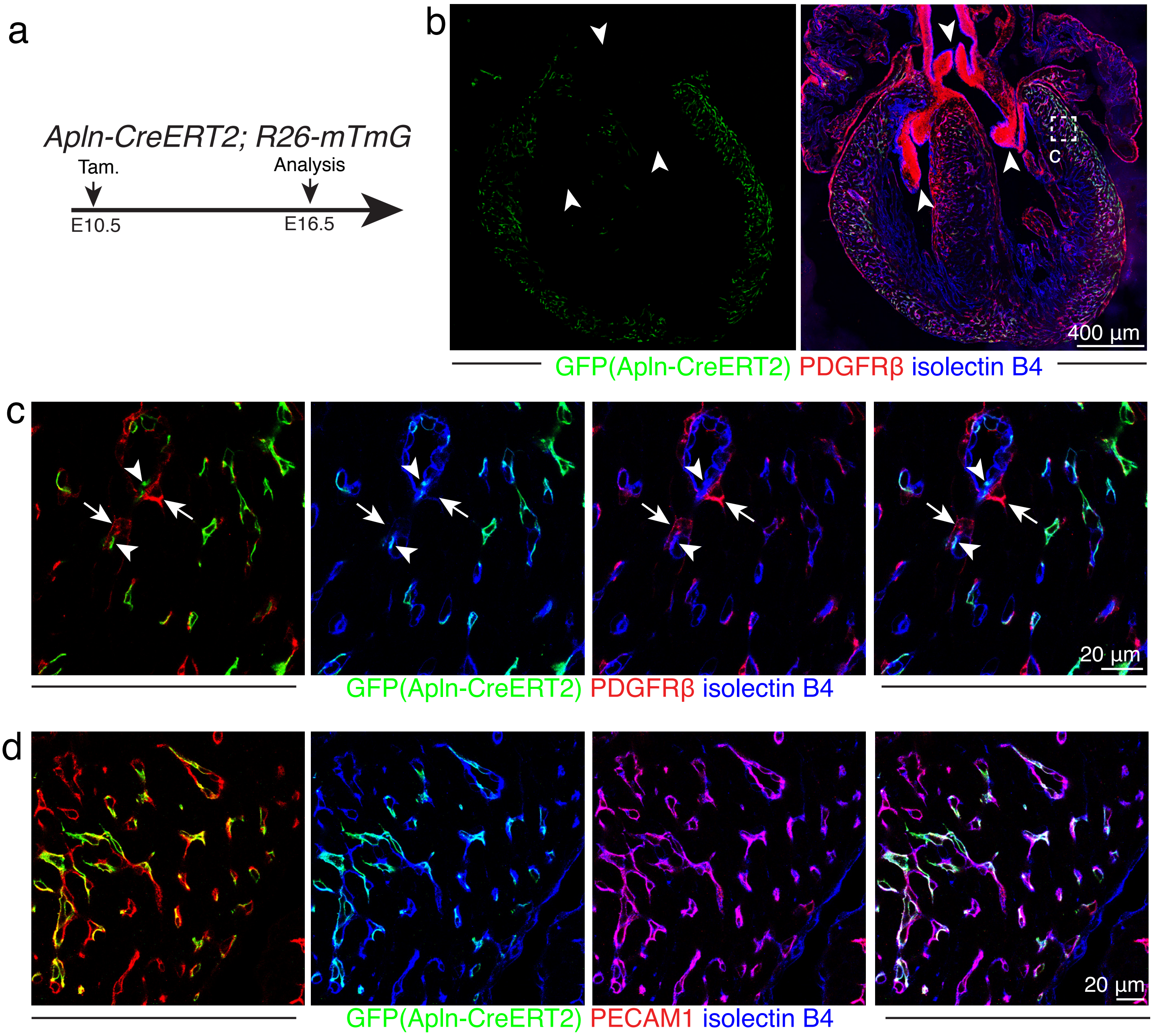

Supplementary Figure 7 I Clonal analysis of subepicardial endothelial cells

(**a**) Clonal analysis strategy of *Apln-CreERT2 Rosa26-mTmG* mice indicating the stage of tamoxifen administration and analysis.

(**b**) At E16.5, GFP signal was found in myocardium and ventricular septum but was absent in PDGFRβ+ mesenchymal cells in heart valves (white arrowheads).

(**c**) Higher magnification images show *Apln-CreERT2*-labeled coronary ECs (white arrowheads), which were distinct from PDGFRβ+ cells (white arrows) in E16.5 myocardium.

(**d**) Higher magnification images showing that *Apln-CreERT2*-labeled cells were coronary ECs, which were stained by PECAM1 and isolectin B4.

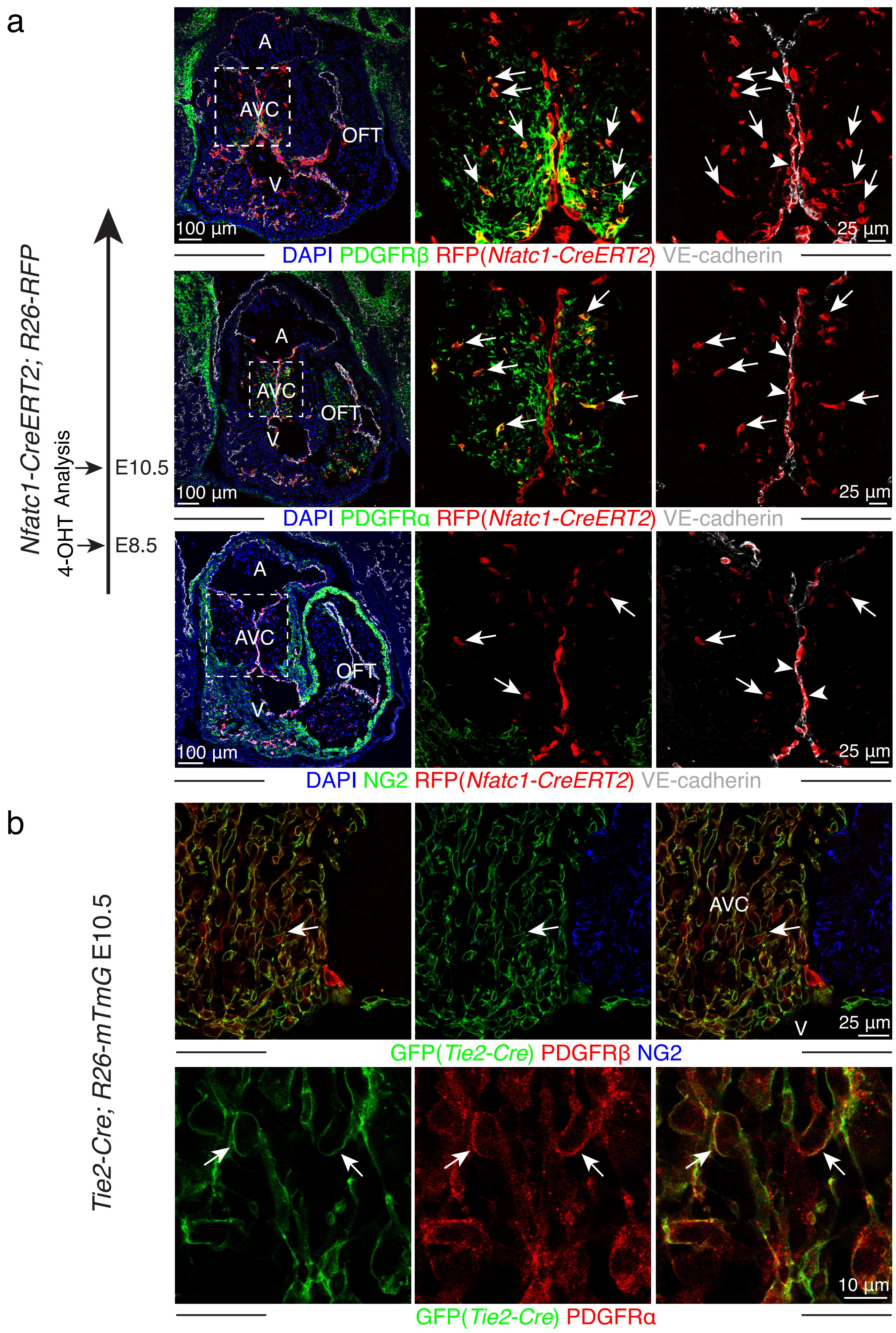
a
A
AVC
OFT
V
100 µm
25 µm
DAPI PDGFRβ RFP(Nfatc1-CreERT2) VE-cadherin
DAPI PDGFRα RFP(Nfatc1-CreERT2) VE-cadherin
DAPI NG2 RFP(Nfatc1-CreERT2) VE-cadherin
Nfatc1-CreERT2; R26-RFP
4-OHT Analysis
E10.5
E8.5
b
Tie2-Cre; R26-mTmG E10.5
AVC
V
25 µm
GFP(Tie2-Cre) PDGFRβ NG2
10 µm
GFP(Tie2-Cre) PDGFRα

Supplementary Figure 8 l Endocardial ECs differentiate into mesenchymal progenitors.
(**a**) Clonal analysis in *Nfatc1-CreERT2 Rosa26-RFP* double transgenic hearts at E10.5 following 4-OHT administration at E8.5. Recombined, RFP+ cells were located in the E10.5 VE-cadherin+ (white) endocardium (arrowheads). RFP+ PDGFRβ+ PDGFRα+ but NG2- cells were also detected in the adjacent endocardial cushions (arrows). A, atrium; V, ventricle; AVC, atrioventricular canal; OFT, outflow tract. Nuclei, DAPI (blue).
(**b**) Lineage tracing in *Tie2-Cre Rosa26-mTmG* double transgenic hearts at E10.5. Higher magnification images show GFP+ PDGFRβ+ PDGFRα+ NG2- cells (arrows) in the atrioventricular canal. V, ventricle; AVC, atrioventricular canal

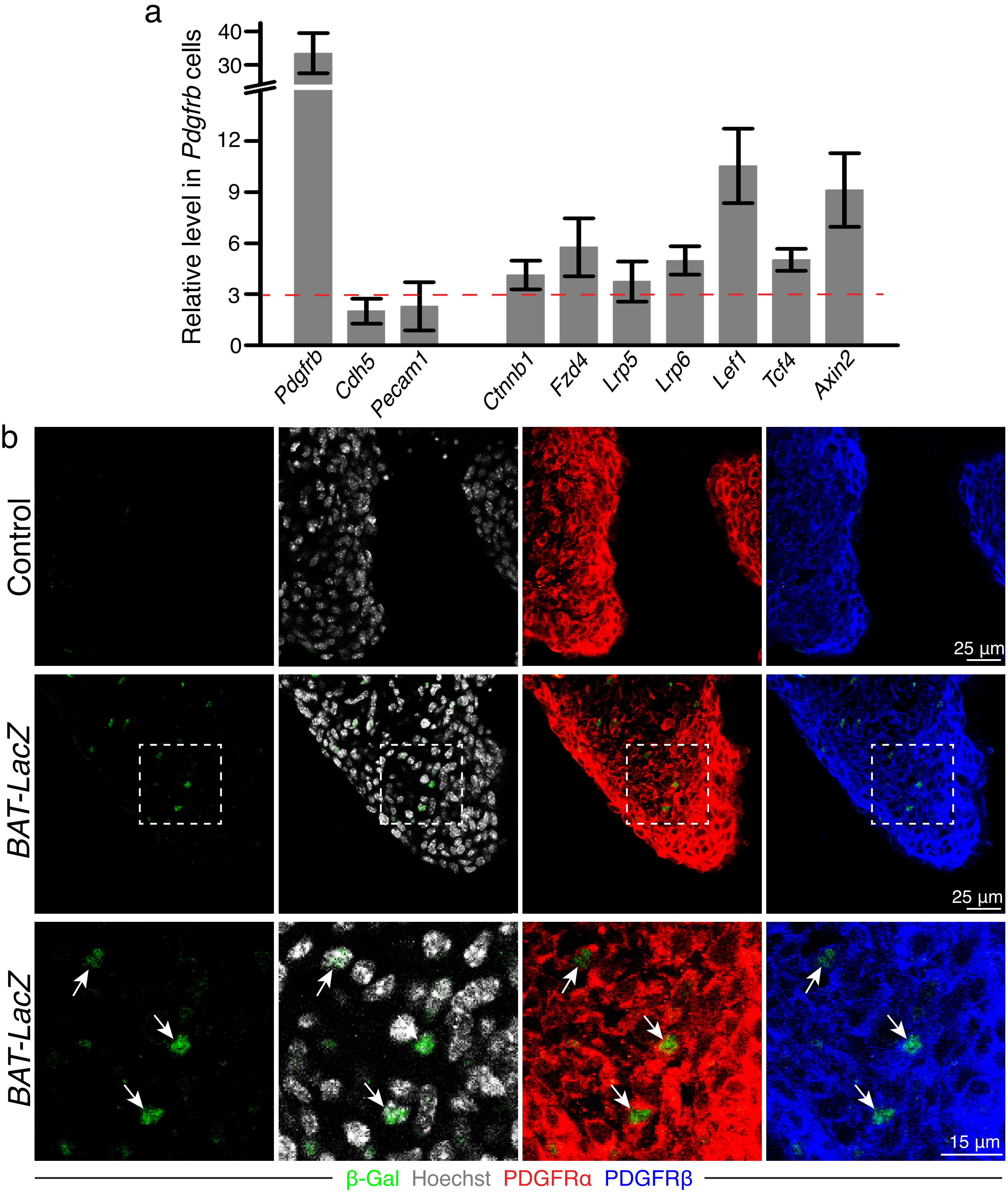

Supplementary Figure 9 | Wnt signaling in embryonic PDGFRβ+ cells in heart.
(**a**) RiboTag RT-qPCR analysis of the indicated Wnt pathway genes in cardiac *Pdgfrb(BAC)-CreERT2*-expressing cells at E13.5. RNAs from RiboTag enrichment fraction were compared with input (See Methods). *Pdgfrb* was used as positive control, *Cdh5* and *Pecam1* as negative controls. n=3 (4 hearts/experiment). Error bars, ± SEM.
(**b**) Detection of Wnt signaling activity with the *Tg(BAT-lacZ)*^{3Picc} reporter (anti-β-galactosidase immunostaining, green) in PDGFRα+ PDGFRβ+ double positive cells of E13.5 heart valves (arrows). No specific signal was detected in littermate control mice lacking the *Tg(BAT-lacZ)*^{3Picc} transgene.

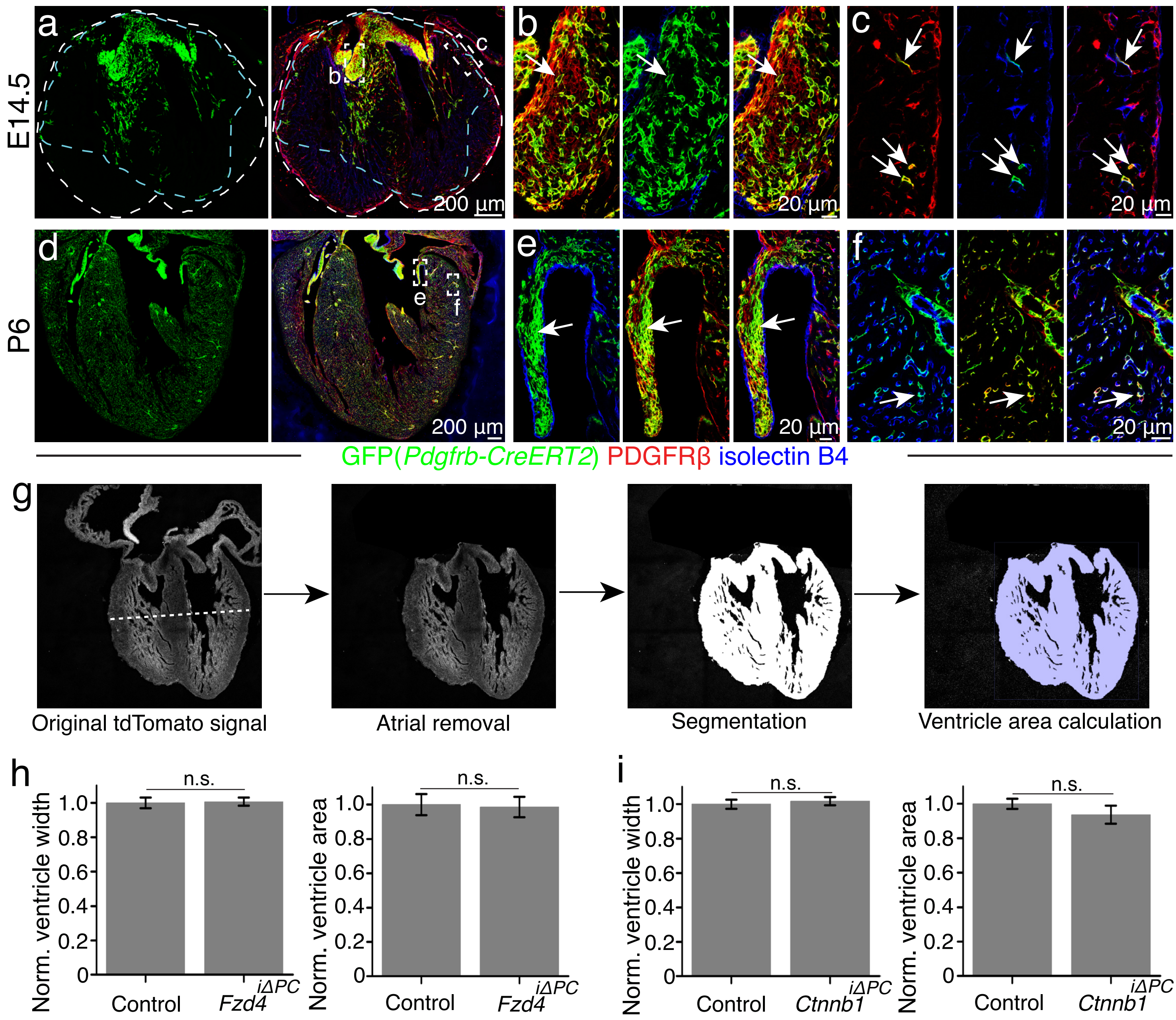

Supplementary Figure 10 I Analysis of Wnt pathway mutant mice.
(**a**-**f**) Distribution of *Pdgfrb(BAC)-CreERT2*-labeled (GFP, green) PDGFRβ+ (red) cells (arrows) at E14.5 (after tamoxifen administration from E9.5 to E12.5; **a**-**c**), and at P6 (tamoxifen from P1-P3; **d**-**f**). Panels on the right show higher magnifications of the corresponding insets with details of heart valves (**b**, **e**) and myocardium (**c**, **f**). ECs, isolectin B4 (blue). White dashed lines in (**a**) mark ventricle size and cyan dashed lines indicate the area containing GFP+ cells, as analyzed in Fig. **6b** and **6d**.
(**g**) Representative images showing the calculation of 'ventricle area' and 'ventricle width' (dashed line in left image), as shown in (**h**) and (**i**). The tdTomato signal of *Rosa26-mTmG* Cre reporter animals was processed and used for calculations, as indicated.
(**h** and **i**) Quantification of ventricle width and area for 8 E14.5 *Fzd4* $^{i\Delta PC}$hearts relative to 8 heterozygous littermate controls (h), and for 7 E14.5 *Ctnnb1*$^{i\Delta PC}$ embryos relative to 8 heterozygous control littermates (i). Error bars, ± SEM. P values, two-tailed unpaired t-test; n.s., no significance.

Supplementary Table 1 | Summary of genetic mouse models used in this study

Full Name	Short name	Inducible	Reporter	Purpose
Pdgfra$^{tm11(EGFP)Sor}$	-		H2B-GFP (nuclear)	Detection of PDGFRα-expressing cells
Tg(Cspg4-dsRed.T1)^{1Akik}	-		DsRed	Detection of NG2/Cspg4-expressing cells
Tg(BAT-lacZ)^{3Picc}	-		β-Gal	Detection of Wnt signaling *in vivo*
Rpl22$^{tm1.1Psam}$	*Rpl22*		HA	Pull-down of actively translating mRNAs
Pdgfrb(BAC)-CreERT2	*Pdgfrb-CreERT2*	Yes	-	Labeling of PDGFRβ+ cells and their descendants
Cdh5(PAC)-CreERT2	*Cdh5-CreERT2*	Yes	-	Labeling of ECs and their descendants
Apln-CreERT2	-	Yes	-	Labeling of subepicardial ECs and their descendants
Nfatc1-CreERT2	-	Yes	-	Labeling of endocardial ECs and their descendants
Tie2-Cre	-		-	Labeling of ECs and their descendants
Wnt1-Cre	-		-	Labeling of neural crest cells and their descendants
Rosa26-mT/mG	*R26-mTmG*		Cre-induced switch from mem-Tomato to mem-GFP	Labeling of Cre-positive cells and their descendants
Rosa26-RFP	*R26-RFP*		RFP	Labeling of Cre-positive cells and their descendants
Fzd4$^{\mathrm{tm2.1Nat}}$	-		-	Conditional knockout of *Fzd4*
Ctnnb1$^{\mathrm{tm2Kem}}$	-		-	Conditional knockout of *Ctnnb1*
Wls$^{\mathrm{tm1.1Lan}}$	-		-	Conditional knockout of *Wls*
Pdgfrb(BAC)-CreERT2 Rosa26-mT/mG	*Pdgfrb-CreERT2 R26-mTmG*	Yes	mem-GFP after Cre recombination	Labeling of PDGFRβ+ cells and their descendants
Cdh5(PAC)-CreERT2 Rosa26-mT/mG	*Cdh5-CreERT2 R26-mTmG*	Yes	mem-GFP after Cre recombination	Labeling of ECs and their descendants
Tie2-Cre Rosa26-mT/mG	*Tie2-Cre R26-mTmG*		mem-GFP after Cre recombination	Labeling of ECs and their descendants
Wnt1-Cre Rosa26-mT/mG	*Wnt1-Cre R26-mTmG*		mem-GFP after Cre recombination	Labeling of neural crest cells and their descendants
Apln-CreERT2 Rosa26-mT/mG	*Apln-CreERT2 R26-mTmG*	Yes	mem-GFP after Cre recombination	Labeling of subepicardial ECs and their descendants
Nfatc1-CreERT2 Rosa26-RFP	*Nfatc1-CreERT2 R26-RFP*	Yes	RFP	Labeling of endocardial ECs and their descendants
Pdgfrb(BAC)-CreERT2 Fzd4$^{\mathrm{tm2.1Nat}}$ *Rosa26-mT/mG*	*Fzd4$^{i\Delta PC}$*	Yes	mem-GFP after Cre recombination	Knockout of *Fzd4* in PDGFRβ+ cells

Pdgfrb(BAC)-CreERT2 Ctnnb1$^{\mathrm{tm2Kem}}$ *Rosa26-mT/mG*	*Ctnnb1*$^{i\Delta PC}$	Yes	mem-GFP after Cre recombination	Knockout of *Ctnnb1* in PDGFRβ+ cells
Cdh5(PAC)-CreERT2 Wls$^{\mathrm{tm1.1Lan}}$	*Wls*$^{i\Delta EC}$	Yes	-	Knockout of *Wls* in ECs
Pdgfrb(BAC)-CreERT2 Rpl22$^{tm1.1Psam}$	*Pdgfrb-CreERT2 Rpl22*	Yes	HA	Pull-down of actively translating mRNAs from PDGFRβ+ cells
